# The estimated dietary and health impact of implementing the recently approved ‘high in’ front-of-package nutrition symbol in Canada: a food substitution scenario modeling study

**DOI:** 10.3389/fnut.2023.1158498

**Published:** 2023-08-07

**Authors:** Nadia Flexner, Mavra Ahmed, Christine Mulligan, Jodi T. Bernstein, Anthea K. Christoforou, Jennifer J. Lee, Neha Khandpur, Mary R. L’Abbe

**Affiliations:** ^1^Department of Nutritional Sciences, Temerty Faculty of Medicine, University of Toronto, Toronto, ON, Canada; ^2^Joannah and Brian Lawson Centre for Child Nutrition, Temerty Faculty of Medicine, University of Toronto, Toronto, ON, Canada; ^3^Department of Human Nutrition and Health, Wageningen University, Wageningen, Netherlands; ^4^Center for Epidemiological Research in Nutrition and Health, School of Public Health, University of São Paulo, São Paulo, Brazil; ^5^Department of Nutrition, Harvard T.H. Chan School of Public Health, Harvard University, Boston, MA, United States

**Keywords:** front-of-pack nutrition label, dietary intakes, diet-related NCD, NCD and risk factors, macrosimulation model, food policy, food substitution

## Abstract

**Background:**

Front-of-pack labeling (FOPL) has been identified as a cost-effective policy to promote healthy food environments and to help consumers make healthier food choices. Consumer surveys report that after implementation of mandatory ‘high in’ FOPL symbols between 30 and 70% of consumers choose or were willing to choose products with fewer ‘high in’ symbols. Health Canada has recently published FOPL regulations that will require prepackaged food and beverages that meet or exceed thresholds for sodium, total sugars, or saturated fat to display a ‘high in’ FOPL nutrition symbol.

**Objectives:**

The aims were to estimate the potential (1) dietary impact of substituting foods with similar foods that would display at least one less ‘high in’ symbol, and (2) the number of diet-related noncommunicable disease (NCD) deaths that could be averted or delayed due to estimated dietary changes.

**Methods:**

Baseline and counterfactual intakes of sodium, total sugars, saturated fats, and energy were estimated among Canadian adults (*n* = 11,992) using both available days of 24 h-recall data from the *2015 Canadian Community Health Survey-Nutrition (CCHS)*. Similar foods to those reported in CCHS that would display at least one less ‘high in’ symbol (*n* = 239) were identified using a Canadian branded food composition database. Based on current FOPL consumer research, identified foods were substituted for 30, 50, and 70% of randomly selected *CCHS-Nutrition* adult participants and for all adult participants. Potential health impacts were estimated using the Preventable Risk Integrated ModEl.

**Results:**

Mean dietary reductions of between 73 and 259 mg/day of sodium, 2.0 and 6.9 g/day of total sugars, 0.2 and 0.5 g/day of saturated fats, and 14 and 46 kcal/day of energy were estimated. Between 2,148 (95% UI 1,913–2,386) and 7,047 (95% UI 6,249–7,886) of deaths due to diet-related NCDs, primarily from cardiovascular diseases (70%), could potentially be averted or delayed if Canadians choose products with fewer ‘high in’ symbols.

**Conclusion:**

Results suggest that FOPL could significantly reduce sodium and total sugar intakes among Canadian adults, the consequences of which could avert or delay an important number of diet-related NCD deaths. These findings provide relevant data to support the importance of the impending FOPL regulations.

## 1. Introduction

Front-of-pack labeling (FOPL) has been identified as a cost-effective policy to promote healthy food environments and to help consumers make healthier food choices (1). Mandatory nutrient warning FOPL systems (‘high in’ or ‘excess’) have been adopted in Argentina (2), Brazil (3), Chile (4), Colombia (5), Israel (6), Mexico (7), Peru (8), Uruguay (9), Venezuela (10), and more recently in Canada (11, 12).

As part of the *Healthy Eating Strategy* (2016), Health Canada highlighted the need to provide consumers with simple and easy to understand nutrition labeling to help Canadians make healthier and informed food choices, specifically on packaged foods that are ‘high in’ nutrients to limit (i.e., total sugars, sodium and saturated fats) (13). In 2018, during public consultations, Health Canada proposed the implementation of a mandatory ‘high in’ FOPL system in *Canada Gazette I* that would require packaged foods that meet or exceed predetermined thresholds for total sugars, sodium, and saturated fats to display a ‘high in’ nutrition symbol on the front of the package (14). Recently, in July 2022, Health Canada published final FOPL regulations in *Canada Gazette II* that will come into effect in January 2026 (11). Thresholds for foods that will be subject to a ‘high in’ FOPL symbol, as well as exemptions to the policy were updated in the finalized regulations (11).

Chile was the first country to implement a ‘high in’ FOPL system in 2016 (Law 20.606) (4). This comprehensive law not only requires foods meeting established thresholds for energy and key critical nutrients (i.e., saturated fats, sodium, and sugars) to display a ‘high in’ FOPL symbol, but also restricts sales and promotion in schools as well as marketing to children of ‘high in’ labeled products (4). Promising first evaluations of this initiative showed significant reductions in the energy, sugar, sodium, and saturated fat content of households’ food and beverage purchases (15); industry-driven food reformulation (16–19); and significant declines in children’s exposure to unhealthy food advertising (20). Nationally representative consumer surveys showed that at least 30% of respondents declared choosing foods with fewer ‘high in’ symbols a year after the implementation of the Law (2017), and in subsequent surveys the percentage was of 58% (2018) and 72% (2019) (21–23).

Similarly, early evaluations of Israel’s FOPL reform showed that most consumers approved and understood the goals of the reform (92.6%) and declared that they would purchase fewer food products with warning labels (81.7%) (24). Moreover, a year after its implementation almost 60% of consumers reported using the FOPL to some degree, and 70% declared their willingness to choose healthier alternatives in the coming year (6).

In Canada, a study showed that in experimental conditions, consumers used the ‘high in’ FOPL symbol to compare foods within the same food category (70%), to choose foods that did not display a ‘high in’ symbol (69%), and to choose healthier alternatives when available (40%) (25), similar to other studies (9, 26). Additionally, the study suggests that with increased awareness and exposure to the ‘high in’ symbol, consumers became more efficient at selecting healthier food alternatives (25).

The sum of the literature to date indicates that recently implemented polices that are similar to the Canadian ‘high in’ FOPL policy have proven to help consumers make healthier food choices (15, 27) and to promote healthy food environments (16–20, 28). However, the long-term impacts on diets and health have yet to be determined given the nascence of these policies. Traditional epidemiological research methods such as cohort studies or randomized control trials are not always feasible for ethical or practical reasons, and often do not provide timely evidence needed for policymaking in public health (29, 30). Therefore, approaches such as policy scenario modeling can address this gap by providing estimates of the potential dietary and health impacts of implementing a ‘high in’ FOPL. Policy scenario modeling is an effective tool in the policymaking process, as it can provide estimated impacts of the policy of interest, using the best available evidence, before actual policy adoption and implementation (29–31).

Previous Canadian studies explored the potential dietary and health impact of substituting foods labeled with at least one red-light label under the United Kingdom’s criteria for traffic light labelling (TLL), with a similar healthier alternative without any red-light label (32, 33). A recent study estimated the potential dietary and health impact of implementing a FOPL in Canada by modeling consumers’ food purchase behavior changes observed in experimental and observational studies (34).

However, to the best of our knowledge, this is the first study exploring potential dietary and health outcomes of substituting foods Canadians consume with similar healthier alternatives under the finalized ‘high in’ FOPL regulations in Canada.

Therefore, the aims of this study were two-fold: (1) to estimate the potential dietary impact of substituting packaged foods actually consumed by Canadians, with similar packaged foods that would display at least one less ‘high in’ symbol, and (2) to estimate the number of diet-related noncommunicable diseases (NCDs) deaths that could be averted or delayed due to the estimated dietary impact of food substitution.

## 2. Materials and methods

### 2.1. Baseline dietary data: *CCHS-Nutrition 2015*

The *Canadian Community Health Survey (CCHS)-Nutrition 2015 Public Use Microdata File (PUMF)* data (35, 36) was used in this study to estimate current sodium, total sugars, saturated fats, fiber, and energy intakes (baseline scenario) among Canadian adults (≥19 y). *CCHS-Nutrition 2015* is a cross-sectional, nationally representative sample survey, conducted by Statistics Canada, that uses 24-h (24 h) dietary recalls to gather information on food and beverage intake across Canada (aged 1 y or older residing in the 10 Provinces of Canada). Canadians residing in the territories, on reservations and other indigenous settlements, full-time members of the Canadian Armed Forces, and institutionalized individuals were excluded from the survey (36).

A modified version of the United States Department of Agriculture (USDA) 5-step Automated Multiple-Pass method was used in *CCHS-Nutrition 2015* given its documented strength in capturing dietary intakes with less misreporting bias (37). *CCHS-Nutrition 2015* was conducted via computer-assisted in-person interviews by trained professionals (*n* = 20,487). The initial 24 h recall was conducted during these in-person interviews. The second 24 h recall was conducted within the next 3–10 days after the first recall, which involved 35% of the total sample, and it was completed via telephone (36). Both available days of 24 h dietary recalls were used in this study to estimate usual dietary intakes for all adults (≥19 y) and by Dietary Reference Intakes (DRI) age-sex groups (38). Health Canada’s Canadian Nutrient File (CNF), version 2015 was used (39) in this study to obtain energy and nutrient content of all foods reported in *CCHS-Nutrition 2015*.

Canadian adults (≥19 years; *n* = 13,919) were included in this analysis, excluding breastfeeding females (*n* = 188), respondents who did not report any food consumption (*n* = 4), underweight respondents (BMI < 18.5 kg/m^2^) and respondents without self-reported or measured height and weight (*n* = 1,735). After exclusions, our final sample was of 11,992 *CCHS-Nutrition 2015* respondents (40). To adjust for dietary misreporting status, energy intake to total energy expenditure ratio was estimated for each respondent, methods that have been described previously (40).

### 2.2. Mean height and BMI data: *CCHS-Nutrition 2015*

Mean height and body mass index (BMI) were estimated by DRI age-sex groups for the final sample. Previous studies using both 2004 and 2015 cycles of *CCHS-Nutrition* have suggested that adult males have the tendency to over-report their height and adult females have the tendency to under-report their weight. Thus, BMI correction factors provided by Statistics Canada were used in this analysis to calculate participants’ height and BMI from self-reported values if no measurements were available, this to lessen the introduction of systematic biases into the analyses (41). The final mean heights and BMI values for the sample were estimated from both corrected and measured values (Supplementary Table S1). BMI and height values were used as inputs for the NCD scenario model used in this study (detailed in section 2.7).

### 2.3. *CCHS-Nutrition 2015* Food and Ingredients Detail (FID) file

The FID file provides nutrient information for individual food and ingredients (*n* = 2,784) reported by all *CCHS-Nutrition 2015* participants. To obtain food composition data (energy and nutrient content) of all foods reported in *CCHS-Nutrition 2015*, we used information from Health Canada’s CNF, version 2015 (39). The CNF provides nutrient composition data for food products commonly consumed in Canada (i.e., fresh, packaged, and prepared foods and beverages) (39). These files and detailed information on them are publicly available (35, 36, 39).

### 2.4. University of Toronto’s Food Label Information and Price (FLIP) database

Food label data from the University of Toronto’s Food Label Information and Price (FLIP) 2017 database were used for this study. Briefly, FLIP 2017 is comprised of Canadian food package label information by brand name for the main foods and beverages sold in Canada. FLIP 2017 contains information on Nutrition Facts table (NFt), ingredients list, product price, barcodes, and photos of all sides of the product packaging for 17,671 unique prepacked food and beverages. Data were collected from the largest grocery retailers in Canada, representing approximately 70% of Canadian grocery retail sales. Data collection was conducted between July and September 2017 using the FLIP mobile data collector app, methods that have been previously described (42).

### 2.5. Identifying healthier alternatives in the Canadian food supply to foods consumed by Canadians

All food products in the FID file and FLIP 2017 were categorized into Health Canada’s Table of Reference Amounts (TRA) for food categories to facilitate comparisons and matching of foods (43). Health Canada’s TRA categories consist of 23 major and 171 minor categories (43). Food and beverage products in FLIP 2017 were matched to FID file foods through a systematic process, methods that have been detailed elsewhere (44). Overall, it was possible to match 56.1% (*n* = 1,561) unique FID foods to one or more FLIP 2017 products (*n* = 15,142), mainly because FLIP 2017 is comprised mostly of packaged foods and does not contain data on unprocessed meat, fish, poultry, and fresh fruit and vegetables. It is worth noting, however, that most of the foods that are not included in FLIP would be exempted from the ‘high in’ FOPL regulations in Canada and would not have been involved in the modeled food substitution scenarios. Then, all foods in both datasets (FID file and FLIP 2017) were assessed under final FOPL regulations published in *Canada Gazette II* (11), methods that have been previously published (45).

To facilitate analysis, data manipulation, and identifying healthier alternatives, datasets were linked by common codes (FID CDE and FLP ID) using R studio version 4.2.2. Healthier alternatives, displaying at least one less ‘high in’ symbol, to products reported in *CCHS-Nutrition 2015* were then identified among similar FLIP 2017 foods categorized under the same TRA minor categories. Foods exempted from FOPL regulations, foods with no healthier alternative, and foods with missing nutrient values were removed (*n* = 13,661). Aggregated nutrient values of healthier alternatives found in FLIP were then calculated for food substitution.

### 2.6. Food substitution counterfactual scenarios

For this analysis, FOPL counterfactual scenarios were based on data reporting consumer behavior change from early evaluations of ‘high in’ FOPL regulations in Chile and Israel (6, 21–24), and from a Canadian retail experiment study (25). Specifically, we looked at the proportion of consumers who, after comparing ‘high in’ FOPL symbols, chose a product with less or zero ‘high in’ symbols [Canada: 69% (25); Chile: 30, 58, and 72% (21–23)], or declared their willingness to choose healthier alternatives [Israel: 70% (6)].

It was assumed that after comparing ‘high in’ symbols between similar packaged foods (same food category) some consumers would choose a healthier packaged alternative that would display at least one less ‘high in’ symbol. Based on FOPL evidence detailed above, we randomly selected 30% (Scenario 1), 50% (Scenario 2), and 70% (Scenario 3) of *CCHS-Nutrition 2015* adult participants who reported consuming at least one product that would display a ‘high in’ symbol under final Health Canada’s FOPL regulations (11), in any of the two 24 h dietary recall days. Additionally, we estimated potential dietary impact of food substitution for 100% of *CCHS-Nutrition 2015* adult participants (Scenario 4).

**Figure 1:**
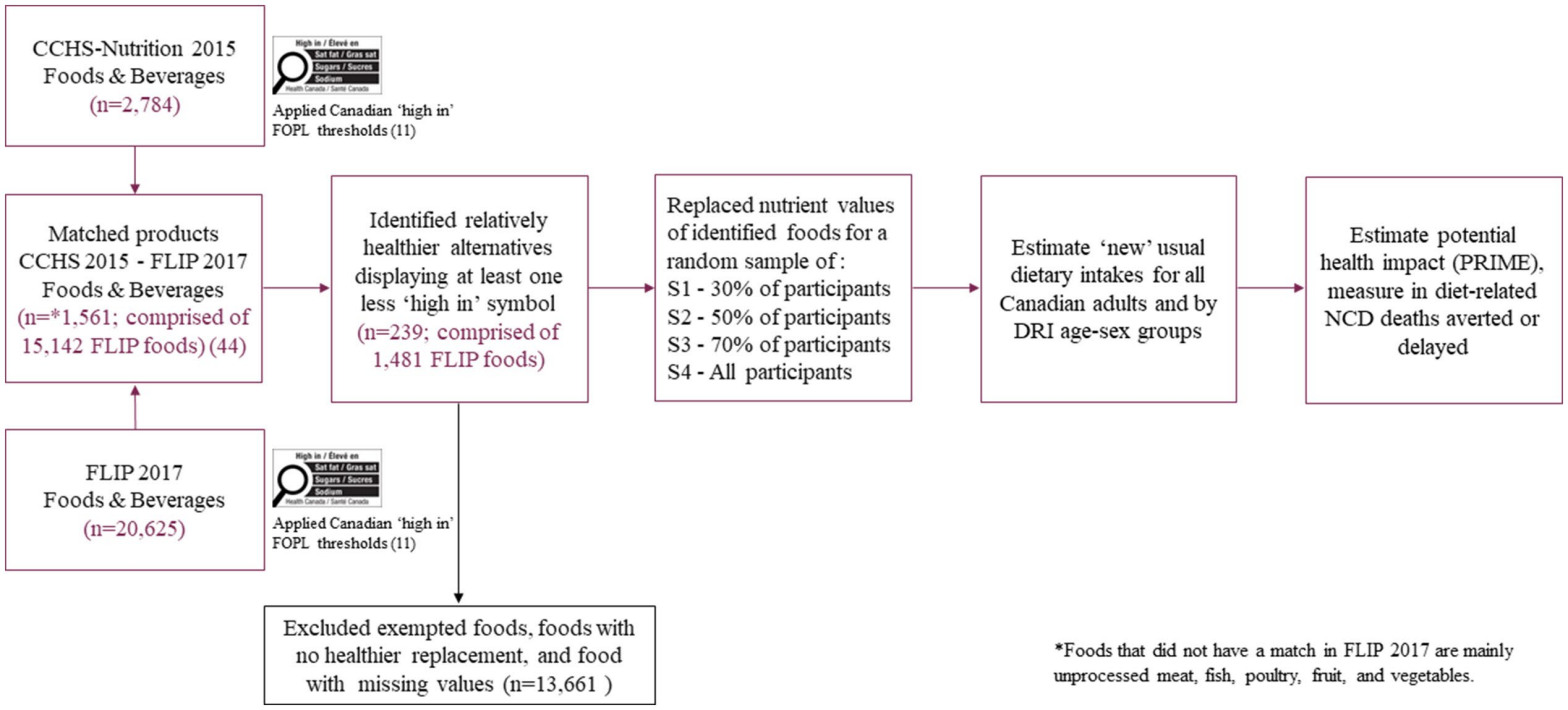
Pathway for modeling the potential dietary and health impact of the Canadian ‘high in’ FOPL symbol from substituting foods consumed by Canadian adults (≥19 y) with similar foods that would display at least one less ‘high in’ FOPL symbol. FOPL, front-of-pack labeling; FLIP, Food Label Information and Price; CCHS, Canadian Community Health Survey; PRIME, Preventable Risk Integrated ModEl; S1, Scenario 1; S2, Scenario 2; S3, Scenario 3; S4, Scenario 4.

Food substitution with a healthier alternative (i.e., displaying at least one less ‘high in’ symbol) was conducted in these three randomly selected sub-groups and all adults. Specifically, we replaced nutritional composition of food products consumed by the randomly selected 30%, 50, and 70% of *CCHS-Nutrition 2015* adult participants with the estimated aggregated nutrient values of the healthier alternatives (saturated fats, sodium, total sugars, fiber and energy), maintaining the same food weight. Finally, we combined these data with the rest of *CCHS-Nutrition 2015* respondents to estimate counterfactual usual dietary intakes for all Canadian adults and by DRI age-sex groups (Figure 1).

Of note, fiber is not included in the FOPL regulations; however, it was included in this study as an exploratory analysis. This was done as, evidence suggests that the use of FOPL improves dietary quality overall by reducing intake of nutrients to limit, such as sugars, and increasing intakes of nutrients to encourage, such as fiber (46).

### 2.7. Health impact modeling

This study used The Preventable Risk Integrated ModEl (PRIME) (29), an open access cross-sectional NCD policy scenario model developed by researchers at the University of Oxford and endorsed by the World Health Organization (WHO) Regional Office for Europe (47). This model has been widely used in different settings and for different behavioral risk factors [i.e., alcohol consumption, smoking, physical activity, and diet (energy, fruits & vegetables, fiber, salt, total fat, saturated fat, unsaturated fat and cholesterol consumption)] (29, 48). Total sugars are not included as a dietary risk factor in PRIME; thus, their health outcomes effects are mediated through calorie reduction. PRIME models the effects on 24 health outcomes including cardiovascular diseases (CVDs), cancers, diabetes, kidney disease, chronic obstructive pulmonary disease, and liver disease (29). PRIME methods have been detailed elsewhere (29).

PRIME answers the question “How many deaths would have occurred in the baseline year if the distribution of risk factors had been different? (47).” Using data from robust meta-analyses of epidemiological studies, the model estimates the impact on population NCD mortality due to changes in the distribution of behavioral risk factors at the population-level. For this study, the model estimated the number of diet-related NCD deaths that could be averted or delayed due to changes in energy and nutrient intakes as a result of modeling the food substitution counterfactual scenarios described above.

The following age- and sex-specific data were included as inputs for the model: (1) number of individuals in Canada (2019); (2) annual number of diet-related NCD deaths (2019); (3) BMI and height estimates; (4) baseline estimates of Canadian adults’ energy, sodium, saturated fat [% of total energy (TE)], and fiber intakes; and (5) counterfactual scenario estimates of Canadian adults’ energy, sodium, saturated fat (% of TE), and fiber intakes after modeling food substitution counterfactual scenarios (Supplementary Tables S1, S3, S5, S6).

This study used 2019 population demographics and mortality data associated with diet-related NCDs (CVDs, diabetes, cancer, chronic renal failure, and liver disease) from the publicly available Statistics Canada CANSIM tables – stratified by sex and five-year age band (49–54) (Supplementary Tables S5, S6). Data from 2019 were used with the assumption of no great changes in Canadians’ dietary intakes since the last *CCHS-Nutrition 2015* survey conducted in 2015 (35, 36). Data on diet-related NCD mortality were based on the WHO International Classification of Diseases 10 (ICD 10) (55).

### 2.8. Statistical analyses

This study determined the number and proportions of *CCHS-Nutrition 2015* FID foods with a healthier alternative in the FLIP 2017 database and average number of FLIP products per FID food. Then, nutritional composition for energy (kcal), saturated fats (g), sodium (mg), sugars (g), and fiber (g) were compared between FLIP and FID food profiles using Mann–Whitney *U* tests given that the means of the food profiles were not normally distributed. Differences were considered statistically significant at *p* < 0.05. Statistical analyses were conducted using R studio version 4.2.2.

Canadian adults’ usual energy and nutrient intakes [sodium, total sugars, saturated fat, saturated fat (%TE), and fiber] were estimated using both available 24 h recall days from *CCHS-Nutrition 2015 PUMF* for baseline (current intakes) and all counterfactual scenarios. We used the National Cancer Institute (NCI) method (56) to estimate usual intakes and distributions overall and by DRI age-sex group, and adjusted for age, sex, misreporting status, weekend/weekday, and sequence of recall. As indicated by Davis et al. (57), we used the 1-part (amount only) model as zero consumption of the studied nutrients was <5%. Additionally, this method allows for stratified analysis by DRI age-sex groups and outlier removal for implausible nutrient intakes. To estimate confidence intervals and standard error, the bootstrap balanced repeated replication method (500 replicates) was used. Sample survey weights provided by Statistics Canada were applied to all analyses to ensure nationally representative estimates (36). Meaningful differences between baseline (current intakes) and food substitution counterfactual scenarios intakes were evaluated by using non-overlapping 95th percentile confidence limits of the means between baseline and counterfactual dietary intakes (58, 59). Statistical analyses were conducted using SAS version 9.4.

After inputting all the required data in PRIME, the number of diet-related NCD deaths that could be averted or delayed were estimated for each food substitution counterfactual scenario, overall and disaggregated by sex and each disease of interest. Monte Carlo analysis was performed at 10,000 iterations to estimate 95% uncertainty intervals (UI) around the results (based on 2.5th and 97.5th percentiles), this allowed the epidemiological parameters in PRIME to vary randomly according to the distributions considered in the model (29).

## 3. Results

### 3.1. Sample characteristics

A total of 11,992 (≥19 y) *CCHS-Nutrition 2015* respondents were included in this analysis, 49.9% of them being females, more than 87% reporting having at least high school diploma or high school equivalency certificate, and more than 44% reported having a household income greater than $80,000/year. Additionally, 32.7% of respondents could be classified as having normal-weight, 36.8% overweight and 30.6% obesity, based on self-reported (adjusted) and measured BMI values (Supplementary Table S1).

### 3.2. Healthier alternatives in the Canadian food supply similar to foods consumed by Canadian adults

Almost 33% (*n* = 918) of foods reported in *CCHS-Nutrition 2015* (*n* = 2,784) would display a ‘high in’ symbol for at least one of the targeted nutrients under final FOPL regulations in Canada. We found healthier alternatives for 26% (*n* = 239) of these foods, comprised of aggregated nutrient values of 1,481 FLIP 2017 food products (Table 1).

**Table 1:** Number and proportions of *CCHS-Nutrition 2015* FID foods with a healthier alternative in the FLIP 2017 database (similar foods) and average number of FLIP products per FID food, overall and by major food group (*n* = 2,784 FID foods).

TRA food category^1^	*n*	FID foods with a healthier alternative in FLIP	Healthier alternatives FLIP foods	Average number (range) of FLIP foods per FID food
Bakery products	231	49 (21.2%)	313	6.4 (1–40)
Beverages	98	8 (8.2%)	28	3.5 (1–9)
Cereals and other grain products	134	8 (6.0%)	77	9.6 (1–44)
Dairy products and substitutes	184	29 (15.8%)	307	10.6 (1–40)
Desserts	60	13 (21.7%)	53	4.1 (1–22)
Dessert toppings and fillings	7	0	0	0
Eggs and eggs substitutes	16	0	0	0
Fats and oils	109	19 (17.4%)	129	6.8 (1–31)
Marine and freshwater animals	144	5 (3.5%)	11	2.2 (1–4)
Fruit and fruit juices	267	13 (4.9%)	58	4.5 (1–14)
Legumes	86	1 (1.2%)	2	2 (2)
Meats and substitutes	520	27 (5.2%)	95	3.5 (1–13)
Miscellaneous category	60	0	0	0
Combination dishes	17	6 (35.3%)	24	4 (1–8)
Nuts and seeds	80	0	0	0
Potatoes, sweet potatoes and yams	25	0	0	0
Sauces, dips, gravies and condiments	60	6 (10.0%)	111	18.5 (1–50)
Snacks	48	7 (14.6%)	55	7.9 (1–28)
Soups	153	26 (17.0%)	78	3 (1–21)
Sugars and sweets	97	11 (11.3%)	68	6.2 (1–47)
Vegetables	292	11 (3.8%)	72	6.5 (1–21)
Foods intended solely for children (<4 y)	71	0	0	0
Meal replacements	14	EXC	EXC	EXC
Others	11	EXC	EXC	EXC
Grand total	2,784	239	1,481	6.2 (1–50)

^1^ Major categories were defined as per Health Canada’s Table of Reference Amounts for Foods (43). TRA, Table of Reference Amounts for foods; FID, Food and Ingredient Details; FLIP, Food Label Information and Price.

Healthier alternatives were found for the following food categories: bakery products (*n* = 49); beverages (*n* = 8); cereals and other grain products (*n* = 8); dairy products and substitutes (*n* = 29); desserts (*n* = 13); fats and oils (*n* = 19); marine and freshwater animals (*n* = 5); fruit and fruit juices (*n* = 13); legumes (*n* = 1); meats and substitutes (*n* = 27); combination dishes (*n* = 6); sauces, dips, gravies and condiments (*n* = 6); snacks (*n* = 7); soups (*n* = 26); sugars and sweets (*n* = 11); and vegetables (*n* = 11). No healthier alternatives were found for the following food categories: dessert toppings and fillings; eggs and eggs substitutes; nuts and seeds; potatoes, sweet potatoes and yams; and foods intended solely for children under 4 years of age (Table 1).

### 3.3. Baseline scenario – current usual energy and nutrient intakes

Canadian adults’ usual mean ± SE energy and nutrient intakes were estimated as follows: energy 1889 ± 20 kcal/day, sodium 2,729 ± 33 mg/day, total sugars 86.4 ± 0.9 g/day, saturated fat 22.8 ± 0.6 g/day, saturated fat (%TE) 10.6 ± 0.18%/day, and fiber 17.3 ± 0.2 g/day (Table 2).

**Table 2:** Canadian adults’ (≥19 y) usual mean energy and nutrient intakes compared with estimated mean intakes after each food substitution counterfactual scenario (*n* = 11,992).

Total (≥19 y)	Baseline mean	Scenario 1 FS 30%	*S1 ∆*	Scenario 2 FS 50%	*S2 ∆*	Scenario 3 FS 70%	*S3 ∆*	Scenario 4 FS 100%	*S4 ∆*
Energy (SE) (kcal/d)	1,889 (20)	1,875 (21)	−14	1,865 (20)	−23	1,857 (21)	−32	1,842 (21)	−46
Sodium (SE) (mg/d)	2,729 (33)	2,656 (37)	−73	2,598 (32) ^*^	−131	2,548 (30) ^*^	−182	2,470 (31) ^*^	−259
Sugars (SE) (g/d)	86.43 (0.95)	84.43 (0.91)	−2.01	82.91 (0.91)	−3.52	81.59 (0.90) ^*^	−4.84	79.50 (0.88) ^*^	−6.93
Saturated fat (SE) (g/d)	22.84 (0.56)	22.69 (0.56)	−0.15	22.60 (0.57)	−0.24	22.52 (0.57)	−0.32	22.36 (0.58)	−0.48
Saturated fat % TE (SE) (g/d)	10.56 (0.18)	10.57 (0.18)	0.01	10.58 (0.18)	0.02	10.59 (0.19)	0.03	10.60 (0.19)	0.04
Fiber (SE) (g/d)	17.27 (0.22)	17.28 (0.21)	0.01	17.27 (0.20)	0	17.25 (0.20)	−0.01	17.24 (0.19)	−0.02

Baseline and counterfactual energy and nutrient intakes were estimated using *CCHS-Nutrition 2015 PUMF* data (35, 36). Usual intakes were estimated using the National Cancer Institute (NCI) method (56), and analyses were adjusted for age, sex, dietary misreporting status, weekend/weekday, and sequence of dietary recall. Usual intakes by DRI age-sex groups are presented in Supplementary Table S3. ^*^Indicates a statistically significant difference between baseline mean intakes and counterfactual mean intakes. Baseline and counterfactual scenarios are described in the methods section. Food substitution was performed for 30% (S1), 50% (S2), and 70% (S3) of randomly selected *CCHS-Nutrition 2015* adult participants and for all adult participants (S4) who consumed at least one packaged food product that would display a ‘high in’ FOPL symbol under Canadian FOPL regulations. d, day; g, grams; SE, standard error; FS, Food Substitution; TE, Total Energy; ∆, Difference; S1, Scenario 1; S2, Scenario 2; S3, Scenario 3; S4, Scenario 4.

### 3.4. FOPL food substitution counterfactual scenarios – potential dietary and health gains

#### 3.4.1. Scenario 1 (S1): food substitution for 30% of randomly selected *CCHS-Nutrition 2015* adult participants

**Figure 2:**
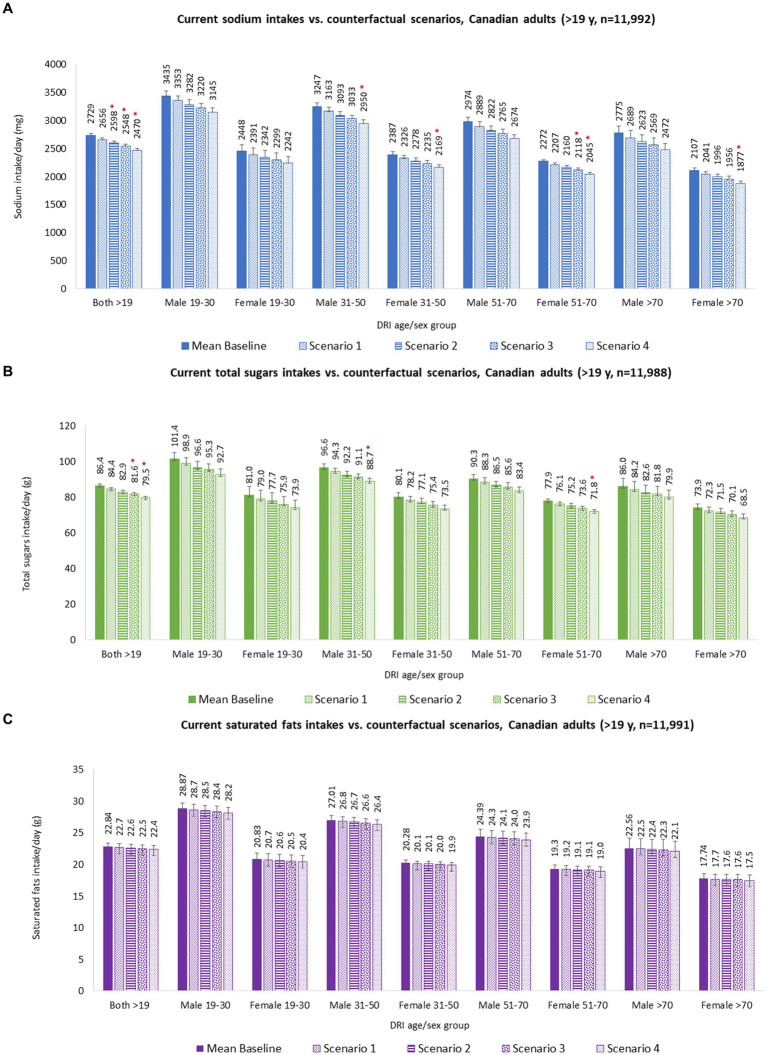

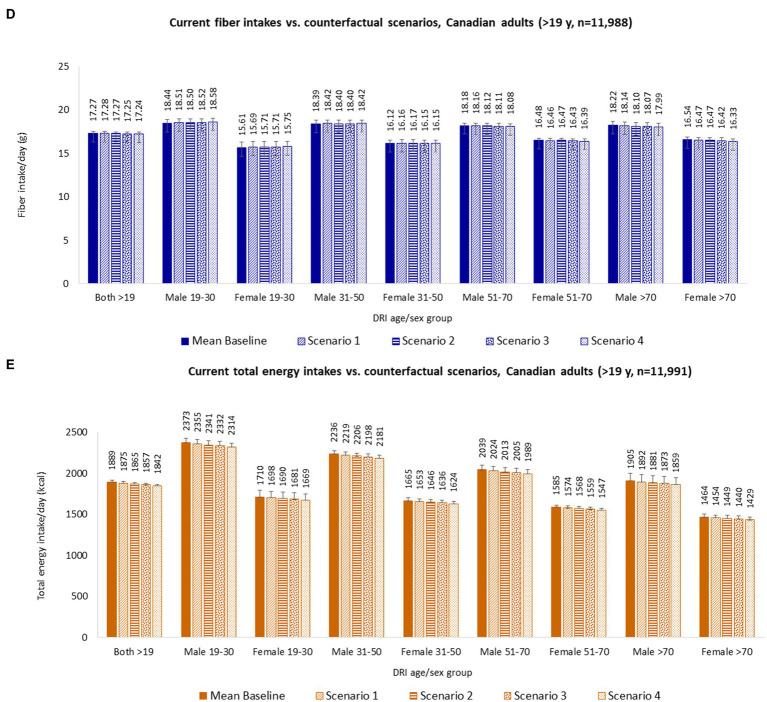
Calorie and nutrient intakes of Canadian adults (≥19 y), overall and by DRI age-sex groups, baseline and counterfactual scenarios. **(A)** Sodium intakes; **(B)** Total sugars intakes; **(C)** Saturated fats intakes; **(D)** Fiber intakes; and **(E)** Total energy intakes. Baseline and counterfactual energy and nutrient intakes were estimated using *CCHS-Nutrition 2015 PUMF* data (35, 36). Usual intakes were estimated using the National Cancer Institute (NCI) method (56), and analyses were adjusted for age, sex, dietary misreporting status, weekend/weekday, and sequence of dietary recall. ^*^Indicates a statistically significant difference between baseline mean intakes and counterfactual mean intakes. Baseline and counterfactual scenarios are described in the methods section. Food substitution was performed for 30% (Scenario 1), 50% (Scenario 2), and 70% (Scenario 3) of randomly selected *CCHS-Nutrition 2015* adult participants and for all adult participants (Scenario 4) who consumed at least one packaged food product that would display a ‘high in’ FOPL symbol under Canadian FOPL regulations. Abbreviations: d = day; g = grams; mg = milligrams; kcal = kilocalories.

Substituting identified healthier food alternatives for a random sample of 30% *CCHS-Nutrition 2015* adult participants resulted in absolute mean dietary reductions of 73 mg/day of sodium, 2.0 g/day of total sugars, 0.2 g/day of saturated fats, and 14 kcal/day of energy for adults overall. For fiber, it resulted in an absolute mean dietary increase of 0.01 g/day. No significant differences were observed between baseline and food substitution counterfactual S1 (Table 2). Stratified dietary intakes were also estimated by DRI age-sex group (Figure 2 and Supplementary Table S3).

**Figure 3:**
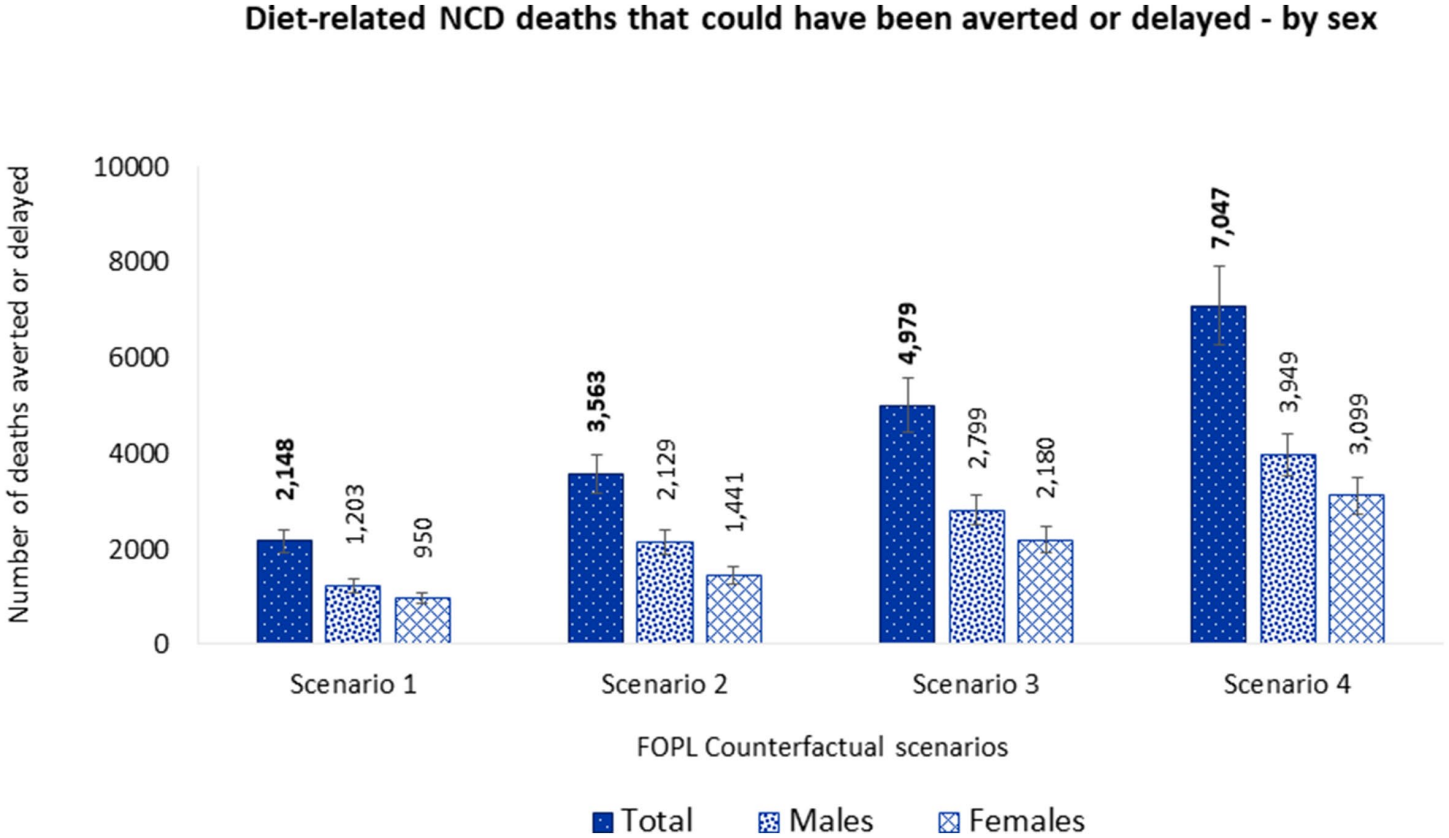
Number of diet related NCD deaths that could be averted or delayed from substituting nutrient composition of packaged foods consumed by Canadian adults (≥19 y) with similar foods that would display at least one less ‘high in’ FOPL symbol – by sex. Potential diet-related NCD deaths that could be averted or delayed were estimated using the PRIME model (29). Inputs for the model included, (1) population demographics; (2) mortality data associated with diet-related NCDs (CVDs, diabetes, cancer, chronic renal failure, and liver disease) (2019), obtained from the publicly available Statistics Canada CANSIM tables (stratified by sex and five-year age band) (49–54); and (3) baseline and counterfactual dietary intakes estimations using *CCHS-Nutrition 2015 PUMF data* (35, 36). Food substitution was performed for 30% (Scenario 1), 50% (Scenario 2), and 70% (Scenario 3) of randomly selected *CCHS-Nutrition 2015* adult participants and for all adult participants (Scenario 4) who consumed at least one packaged food product that would display a ‘high in’ FOPL symbol under Canadian FOPL regulations. NCD, noncommunicable disease; FOPL, front-of-pack labeling.

Overall, estimated energy and nutrient intake changes could potentially avert or delay 2,148 (95% UI 1,913–2,386) deaths from diet-related NCDs, with 56% of these estimated in males (1,203 [95% UI 1,064–1,343]) and 44% in females (950 [95% UI 832–1,068]) (Figure 3 and Supplementary Table S4.1).

**Figure 4:**
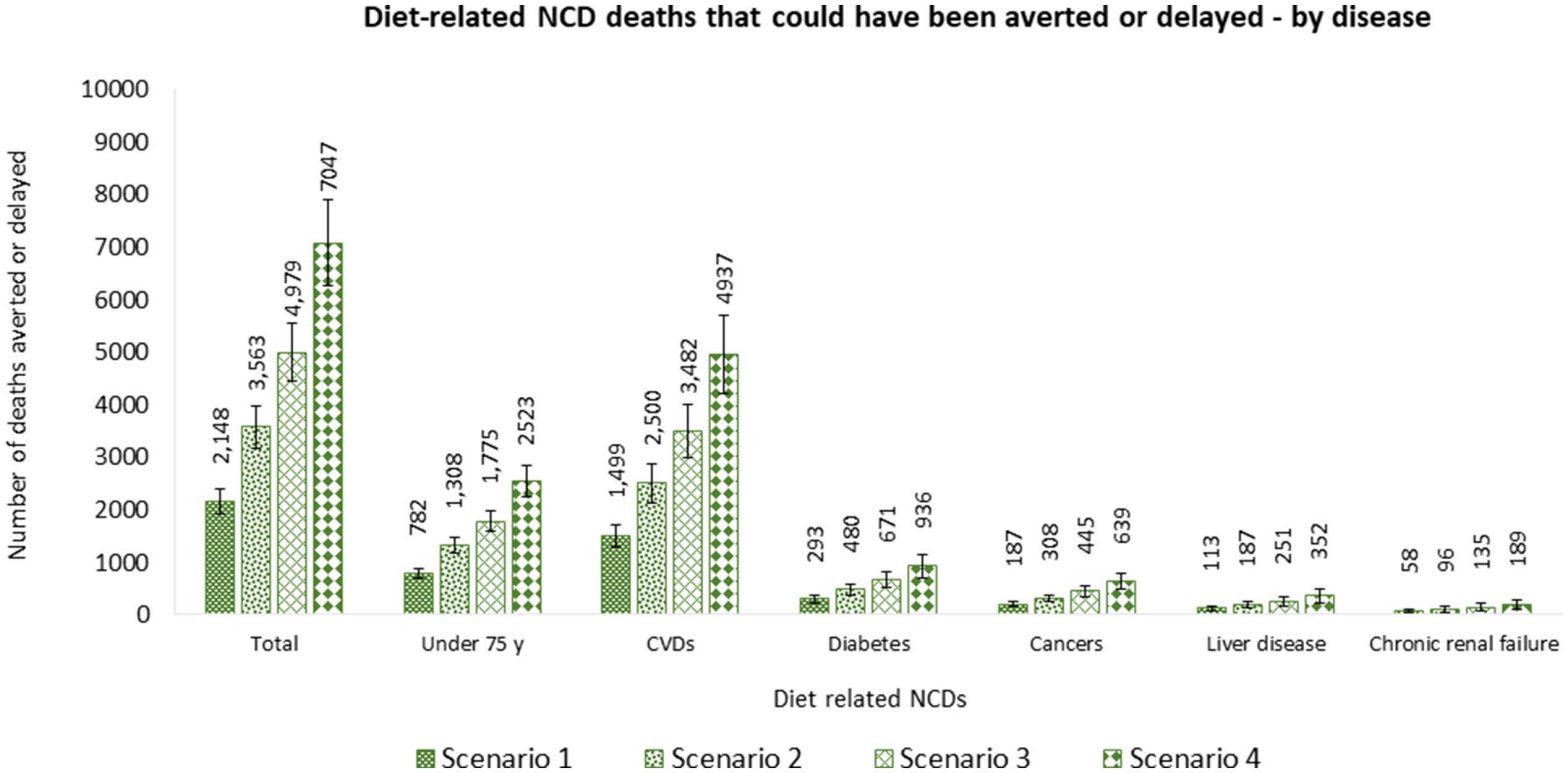
Number of diet related NCD deaths that could be averted or delayed from substituting nutrient composition of packaged foods consumed by Canadian adults (≥19 y) with similar foods that would display at least one less ‘high in’ FOPL symbol – by cause of death. Potential diet-related NCD deaths that could be averted or delayed were estimated using the PRIME model (29). Inputs for the model included, (1) population demographics; (2) mortality data associated with diet-related NCDs (CVDs, diabetes, cancer, chronic renal failure, and liver disease) (2019), obtained from the publicly available Statistics Canada CANSIM tables (stratified by sex and five-year age band) (49–54); and (3) baseline and counterfactual dietary intakes estimations using *CCHS-Nutrition 2015 PUMF data* (35, 36). Food substitution was performed for 30% (Scenario 1), 50% (Scenario 2), and 70% (Scenario 3) of randomly selected *CCHS-Nutrition 2015* adult participants and for all adult participants (Scenario 4) who consumed at least one packaged food product that would display a ‘high in’ FOPL symbol under Canadian FOPL regulations. NCD, noncommunicable disease; FOPL, front-of-pack labeling.

Of the total diet-related NCD deaths that could be averted or delayed, 69.8% (1,499 [95% UI 1,284–1,716]) were related to CVDs, followed by diabetes 13.6% (293 [95% UI 225–353]), cancers 8.7% (187 [95% UI 145–229]), liver disease 5.3% (113 [95% UI 70–154]), and chronic renal failure 2.7% (58 [95% UI 29–88]). Moreover, 36.4% (782 [95% UI 695–866]) of potential deaths averted or delayed would be in people under 75 years old. More lives would be saved in males (545 [95% UI 483–608]) than females (237 [95% UI 208–265]) aged under 75 y (Figure 4 and Supplementary Table S4.1).

#### 3.4.2. Scenario 2 (S2): food substitution for 50% randomly selected *CCHS-Nutrition 2015* adult participants

Substituting identified relative healthier food alternatives for a random sample of 50% *CCHS-Nutrition 2015* adult participants resulted in absolute mean dietary reductions of 131 mg/day of sodium, 3.5 g/day of total sugars, 0.2 g/day of saturated fats, and 23 kcal/day of energy for adults overall. No changes were observed for fiber mean intake. Between baseline and food substitution counterfactual S2, significant differences were observed only for mean sodium intakes (Table 2). Stratified dietary intakes were also estimated by DRI age-sex group (Figure 2 and Supplementary Table S3).

Overall, estimated energy and nutrient intake changes could potentially avert or delay 3,563 (95% UI 3,163–3,969) deaths from diet-related NCDs, with 60% of these estimated in males (2,129 [95% UI 1,886–2,372]) and 40% in females (1,441 [95% UI 1,247–1,625]). Overall, more lives would be saved in males than females (Figure 3 and Supplementary Table S4.2).

Of the total diet-related NCD deaths that could be averted or delayed, 70.2% (2,500 [95% UI 2,122–2,872]) were related to CVDs, followed by diabetes 13.5% (480 [95% UI 370–579]), cancers 8.6% (308 [95% UI 237–377]), liver disease 5.2% (187 [95% UI 117–253]), and chronic renal failure 2.7% (96 [95% UI 45–145]). Moreover, 36.7% (1,308 [95% UI 1,162–1,458]) of potential deaths averted or delayed would be in people under 75 years old. More lives would be saved in males (946 [95% UI 835–1,053]) than females (365 [95% UI 317–411]) aged under 75 y (Figure 4 and Supplementary Table S4.2).

#### 3.4.3. Scenario 3 (S3): food substitution for 70% randomly selected *CCHS-Nutrition 2015* adult participants

Substituting identified relative healthier food alternatives for a random sample of 70% *CCHS-Nutrition 2015* adult participants resulted in absolute mean dietary reductions of 182 mg/day of sodium, 4.8 g/day of total sugars, 0.3 g/day of saturated fats, and 32 kcal/day of energy for adults overall. For fiber, it resulted in an absolute mean dietary decrease of 0.01 g/day. Between baseline and food substitution counterfactual S3, significant differences were observed for mean sodium and total sugar intakes (Table 2). Stratified dietary intakes were also estimated by DRI age-sex group (Figure 2 and Supplementary Table S3).

Overall, estimated energy and nutrient intake changes could potentially avert or delay 4,979 (95% UI 4,446–5,555) deaths from diet-related NCDs, with 56% of these estimated in males (2,799 [95% UI 2,494–3,124]) and 44% in females (2,180 [95% UI 1,912–2,450]). Overall, more lives would be saved in males than females (Figure 3 and Supplementary Table S4.3).

Of the total diet-related NCD deaths that could be averted or delayed, 69.9% (3,482 [95% UI 2,984–3,994]) were related to CVDs, followed by diabetes 13.5% (671 [95% UI 514–808]), cancers 8.9% (445 [95% UI 343–549]), liver disease 5.0% (251 [95% UI 154–345]), and chronic renal failure 2.7% (135 [95% UI 63–204]). Moreover, 35.6% (1,775 [95% UI 1,578–1,978]) of potential deaths averted or delayed would be in people under 75 years old. More lives would be saved in males (1,235 [95% UI 1,094–1,380]) than females (540 [95% UI 474–608]) aged under 75 y (Figure 4 and Supplementary Table S4.3).

#### 3.4.4. Scenario 4 (S4): food substitution for all *CCHS-Nutrition 2015* adult participants

Substituting identified healthier food alternatives for all *CCHS-Nutrition 2015* adult participants resulted in absolute mean dietary reductions of 259 mg/day of sodium, 6.9 g/day of total sugars, 0.5 g/day of saturated fats, and 46 kcal/day of energy for adults overall. For fiber, it resulted in an absolute mean dietary decrease of 0.02 g/day. Between baseline and food substitution counterfactual S4, significant differences were observed for mean sodium and total sugar intakes (Table 2). Stratified dietary intakes were also estimated by DRI age-sex group (Figure 2 and Supplementary Table S3).

Overall, estimated energy and nutrient intake changes could potentially avert or delay 7,047 (95% UI 6,249–7,886) deaths from diet-related NCDs, with 56% of these estimated in males (3,949 [95% UI 3,508–4,403]) and 44% in females (3,099 [95% UI 2,710–3,497]). Overall, more lives would be saved in males than females (Figure 3 and Supplementary Table S4.4).

Of the total diet-related NCD deaths that could be averted or delayed, 70.1% (4,937 [95% UI 4,199–5,705]) were related to CVDs, followed by diabetes 13.3% (936 [95% UI 700–1,129]), cancers 9.1% (639 [95% UI 489–782]), liver disease 5.0% (352 [95% UI 214–483]), and chronic renal failure 2.7% (189 [95% UI 87–285]). Moreover, 35.8% (2,523 [95% UI 2,233–2,822]) of potential deaths averted or delayed would be in people under 75 years old. More lives would be saved in males (1,754 [95% UI 1,549–1,958]) than females (769 [95% UI 672–869]) aged under 75 y (Figure 4 and Supplementary Table S4.4).

To put the presented results in context, in 2019, it was estimated that the total number of diet-related NCD deaths in Canada was 92,845 (males 46,568; females 46,277) (50–54); results from this study would represent 2.3% (2.6% in males, 2.1% in females), 3.8% (4.6% in males, 3.1% in females), 5.4% (6.0% in males, 4.7% in females), and 7.6% (8.5% in males, 6.7% in females) fewer diet-related NCD deaths that could have been averted or delayed under S1, S2, S3 and S4, respectively (Supplementary Table S4). Of the total number of diet-related NCD deaths that could be averted or delayed, ~87% would be attributable to changes in energy intakes (changes in obesity status), and 15% would be attributable to changes in sodium intakes (Table 3).

**Table 3:** Estimated number of diet-related NCD deaths that could be averted or delayed by risk factor.

By risk factor	Scenario 1 (FS 30%)		Scenario 2 (FS 50%)		Scenario 3 (FS 70%)		Scenario 4 (FS 100%)	
	n (95% UI)	%	n (95% UI)	%	n (95% UI)	%	n (95% UI)	%
Saturated fat	−8 (−10, −7)	0	−14 (−17, −11)	0	−19 (−24, −15)	0	−22 (−27, −17)	0
Sodium	320 (136, 508)	15	550 (219, 876)	15	753 (330, 1,191)	15	1,112 (460, 1,803)	16
Energy	1,890 (1,732, 2,048)	88	3,110 (2,849, 3,369)	87	4,369 (4,009, 4,741)	88	6,172 (5,650, 6,695)	88
Fiber	−44 (−63, −26)	−2	−60 (−86, −34)	−2	−84 (−120, −49)	−2	−137 (−194, −79)	−2
Total deaths ^*^	2,148 (1,913, 2,386)	100	3,563 (3,163, 3,969)	100	4,979 (4,446, 5,555)	100	7,047 (6,249, 7,886)	100

Numbers of diet-related NCD deaths that could be averted or delayed were estimated using the PRIME model, 95% UI are based on 10,000 iterations of Monte Carlo analysis built in PRIME (29). Details regarding the number of diet-related NCD deaths for each scenario for each of the NCDs are presented in Supplementary Table S4. ^*^Total deaths averted or delayed represent less than the sum of the individual risk factors given that double counting has been accounted for in PRIME during the modeling process. Food substitution was performed for 30% (Scenario 1), 50% (Scenario 2), and 70% (Scenario 3) of randomly selected *CCHS-Nutrition 2015* adult participants and for all adult participants (Scenario 4) who consumed at least one packaged food product that would display a ‘high in’ FOPL symbol under Canadian FOPL regulations. FS, Food Substitution; UI, Uncertainty Intervals.

## 4. Discussion

This is the first study to estimate the potential dietary and health outcomes of substituting packaged foods consumed by Canadians with similar available healthier alternatives according to the recently published ‘high in’ FOPL regulations in Canada. Current energy and nutrient intakes estimations for Canadian adults presented in this study (baseline scenario) are in line with prior estimations using *CCHS-Nutrition 2015* data (40, 60). Our results suggest that substituting packaged foods that are ‘high in’ nutrients to limit (i.e., saturated fats, sodium, and sugars) with similar healthier food alternatives available in the Canadian food supply (displaying at least one less ‘high in’ symbol), could significantly reduce Canadian adults’ mean intakes of sodium (under S2, S3, and S4) and total sugars (under S3 and S4). These results are meaningful given that excess intakes of sodium and sugars increase risk for many NCDs, such as hypertension, CVDs, diabetes, and renal disease (61–68). Our findings are similar to other policy scenario modeling studies looking at the potential impact on nutrient intakes from substituting foods in the presence of a FOPL as counterfactual scenarios (32, 69, 70). However, we did not find any significant differences for saturated fats and fiber mean intakes.

Estimated dietary changes modeled in this study were only attributable to consumer behavior change, assuming consumers will choose a healthier food alternative in response to the ‘high in’ FOPL symbol, as current evidence suggests (6, 21–25). However, FOPL regulations can also have a positive effect on industry-driven food reformulation, as seen in other countries (16–19, 28), which was not considered in this study. Therefore, our results provide conservative estimates and probably underestimate the impact of the ‘high in’ FOPL symbol in Canada. Additionally, initial industry-driven food reformulation prior to implementation of a ‘high in’ FOPL, as seen in Chile (16–19) and Peru (28), could increase the availability of healthier food alternatives in the food supply, which in turn could result in greater impacts on dietary intakes of nutrients to limit. Even though some of the results under the scenarios modeled in this study may appear modest, they translate into important population-level health gains.

Evidence on FOPL in Canada suggests that consumers will be willing to adopt and use the ‘high in’ symbol. For instance, Canadian consumers showed a preference for a FOPL that highlights individual nutrients of public health concern such as the approved ‘high in’ symbol (71). FOPL has shown to be an effective tool to help Canadians with different levels of health literacy to identify foods high in critical nutrients and to help consumers make healthier food choices (25). Additionally, in the presence of a ‘high in’ FOPL vs. no FOPL, Canadian consumers purchased beverages containing less sugar, saturated fats, and energy, as well as snack foods containing less sodium and energy (72). Adding to this body of evidence, our results show the potential impact of the recently approved Canadian FOPL regulations on reducing intakes of nutrients to limit, which remain at high levels in the diets of Canadians (40, 73).

Using PRIME, it was estimated that a considerable number of diet-related NCD deaths could be averted or delayed in Canada as a result of different degrees (%) of potential food substitution scenarios tested in this study. Similar to previous Canadians’ estimates (33, 34), most of the lives that could be saved are from CVDs in all counterfactual scenarios, followed by diabetes, cancers, liver disease, and chronic renal failure. Given that CVDs are the second leading cause of death in Canada (74), hypertension affects 23% of adults (75), 30% of the population have diabetes or pre-diabetes (76), and obesity and overweight affects 63% of adults (77), our estimates suggests that using FOPL to choose healthier food alternatives has the potential to decrease the burden of the most problematic diet-related NCDs affecting Canadians. It is worth noting that estimated health gains were more meaningful for males than females in diseases such as ischemic heart disease, where the number of deaths that could be averted or delayed for males were twice that estimated for females. This, in part, could be explained by the known differences in nutrient intakes (40, 60) and diet-related NCD mortality burden between Canadians males and females (49–54).

The behavioral risk factor associated with the largest numbers of deaths that could be averted or delayed across all counterfactual scenarios were changes in energy intakes, followed by sodium intakes. Although, our findings provide more conservative estimates for potential reductions in energy intakes when compared with a previous Canadian study (32) that looked at substituting foods for all adults (similar to our tested S4) under the United Kingdom’s traffic light label system (46 kcal/day vs. 106 kcal/day), our results indicate greater impacts on sodium reduction intakes (259 mg/day vs. 182 mg/day) (32). The reasons for these differences could lie in the different methodologies used for identifying healthier alternatives (32), methodological differences between the 2004 and 2015 *CCHS-Nutrition* (e.g., data collection and survey design) (36), different nutrient profiles for certain foods and beverages between the 2004 and 2015 *CCHS-Nutrition* due to updates to the CNF database (36), and in the underlying differences between the nutrient profile models used (Health Canada’s ‘high in’ FOPL symbol vs. United Kingdom’s traffic light label system).

There are several strengths and limitations of this study that should be considered when interpreting our results. This was the first study to estimate the potential dietary and health impacts of the recently approved ‘high in’ FOPL regulations in Canada using data from the nationally representative *CCHS-Nutrition 2015* survey. This study used both 24 h recall days to estimate baseline and counterfactual usual dietary intakes. Robust methodologies to estimate usual dietary intakes, such as the NCI method, were used to assess usual energy and nutrient intakes for all Canadian adults, as well as intakes stratified by DRI age-sex group, which were used as disaggregated inputs for the PRIME model.

For this study, healthier alternatives were identified from a large nationally representative branded food composition database of Canadian packaged foods and beverages (FLIP) collected in 2017, the closest FLIP collection to the *CCHS-Nutrition 2015*. This was done after systematically matching FLIP products to equivalent generic packaged foods from the *CCHS-Nutrition 2015* FID file, novel methods that have been described elsewhere (44). Briefly, when comparing the nutritional composition of FID food profiles with the FLIP 2017 food profiles (aggregated values of matched products), these were not different in most food categories. The one exception was the *meats and substitutes* food category (44). These methods gave us a more realistic food substitution scenario, in terms of nutritional composition and availability of healthier packaged food alternatives in the Canadian food supply at one point in time.

Furthermore, counterfactual scenarios modeled in this study were based on the most recent available evidence of the proportion of consumers choosing food products with fewer nutrient warning FOPL symbols when buying foods at different point of times after policy implementation (6, 21–25). Moreover, we present conservative estimates given that initial industry-driven food reformulation, a consequence of FOPL policies, has not been considered – future studies evaluating the Canadian ‘high in’ FOPL symbol will be needed to update our results when reformulation data become available.

However, our study did not consider other consumer purchasing behaviors that could occur, such as not changing their purchase behavior in response of the ‘high in’ FOPL, abandoning consumption of the product altogether or increasing consumption of fresh produce or minimally processed foods. These scenarios should be explored further when more evidence on FOPL impacts is available.

Regarding the model used in this study, PRIME is a macrosimulation model, widely applied in different contexts (29, 48) that utilizes relative risks from robust meta-analyses (29). However, the model does not consider the effect of a time lag between the exposure and disease outcome; thus, it is not clear how long after the change in risk factor exposure the estimated health gains would occur. Nonetheless, the model allows researchers to estimate the population-level health impact of different NCD policy scenarios, which is key evidence to inform the policymaking process and for prioritization of resources when needed. PRIME strengths and limitations have been discussed in detail elsewhere (29, 48).

Lastly, it is critical to accompany implementation of the recently approved Canadian ‘high in’ FOPL regulations with robust independent evaluation and monitoring systems to measure effectiveness and compliance with the policy, as well as to detect and correct any unintended consequences that could diminish potential dietary and health impacts. Evaluations from other countries that have implemented similar mandatory nutrient warning FOPL approaches should also be considered in Canada. For instance, in Chile, many positive effects have been seen after implementation of the Chilean Food Labeling and Marketing Law (15–20), but also some unintended consequences, such as the significant increase in non-nutritive sweetener intakes among preschoolers (78). Therefore, future evaluations of the recently approved Canadian ‘high in’ FOPL regulations should measure the impact of this policy on food choices, diet quality, and ultimately health outcomes; as well as any unintended consequences from this policy, such as assessing reformulation of food products to contain lower levels of nutrients of public health concern and their possible replacements (e.g., starches or fats), changes in prices of reformulated products, the introduction of new food products, stigma over consumption of certain foods, and the triggering of eating disorders. It will also be necessary to understand how this policy could affect different sub-groups of the population.

## 5. Conclusion

Findings suggest that food substitution with a healthier food alternative, based on the display of fewer ‘high in’ FOPL symbols, could improve dietary intakes, especially with regards to sodium and total sugars intakes among Canadian adults. The consequences of which could avert or delay up to 7,047 diet-related NCD deaths in Canada, primarily from CVDs. Overall, our results show that expected consumer behavior changes in response to the impending FOPL policy will improve Canadians dietary intakes and provide important evidence of the potential impact of implementing the recently approved ‘high in’ FOPL regulations in Canada.

## Data availability statement

Canadian Community Health Survey-Nutrition 2015 Public Use Microdata File (PUMF) data is publicly and freely available without restriction at Statistics Canada, https://www150.statcan.gc.ca/n1/en/catalogue/82M0024X. Analytic code (SAS) can be made available to researchers upon request to the author. Canadian population demographics and data on mortality associated with diet-related NCDs (CVDs, diabetes, cancer, chronic renal failure, and liver disease) — stratified by sex and five-year age band — were obtained from the publicly available Statistics Canada CANSIM tables (50). https://www150.statcan.gc.ca/t1/tbl1/en/tv.action?pid=1710000501; https://www150.statcan.gc.ca/t1/tbl1/en/tv.action?pid=1310014201; https://www150.statcan.gc.ca/t1/tbl1/en/tv.action?pid=1310014401; https://www150.statcan.gc.ca/t1/tbl1/en/tv.action?pid=1310014701; https://www150.statcan.gc.ca/t1/tbl1/en/tv.action?pid=1310015101; https://www150.statcan.gc.ca/t1/tbl1/en/tv.action?pid=1310014801.

## Author contributions

NF, NK, MA, CM, and ML’A conceptualized the study design and interpreted the findings. NF conducted the study, wrote the original draft, and performed the statistical analysis. All authors critically reviewed and approved the final manuscript.

## Funding

This research was funded by Canadian Institutes of Health Research (CIHR) operating grants (PJT-165858; SA2-152805; Healthy Cities Training Award). https://cihr-irsc.gc.ca/e/193.html. The funders had no role in study design, data collection and analysis, decision to publish, or preparation of the manuscript.

## Conflict of interest

The authors declare that the research was conducted in the absence of any commercial or financial relationships that could be construed as a potential conflict of interest.

## Publisher’s note

All claims expressed in this article are solely those of the authors and do not necessarily represent those of their affiliated organizations, or those of the publisher, the editors and the reviewers. Any product that may be evaluated in this article, or claim that may be made by its manufacturer, is not guaranteed or endorsed by the publisher.
