# Supplementary material for: The estimated dietary and health impact of implementing the recently approved ‘high in’ front-of-package nutrition symbol in Canada: a food substitution scenario modeling study
Source: Front Nutr. 2023 Aug 7;10:1158498. doi: 10.3389/fnut.2023.1158498 (PMC10443708; doi:10.3389/fnut.2023.1158498)
Supplement: Supplementary file 1 [file Data_Sheet_1.docx]

**Supplemental Materials**

**The estimated dietary and health impact of implementing the recently approved ‘high in’ front-of-package nutrition symbol in Canada: a food substitution scenario modeling study**

Nadia Flexner^1^, Mavra Ahmed^1,2^, Christine Mulligan^1^, Jodi T. Bernstein^1^, Anthea K. Christoforou^1^, Jennifer J. Lee^1^, Neha Khandpur^3,4,5^, Mary R. L’Abbe^1*^

^1^Department of Nutritional Sciences, Temerty Faculty of Medicine, University of Toronto, Toronto, ON, Canada.

^2^Joannah & Brian Lawson Centre for Child Nutrition, Temerty Faculty of Medicine, University of Toronto, Toronto, ON, Canada.

^3^ Department of Human Nutrition and Health, Wageningen University, Wageningen, the Netherlands.

^4^Center for Epidemiological Research in Nutrition and Health, School of Public Health, University of São Paulo, São Paulo, Brazil.

^5^Department of Nutrition, Harvard T. H. Chan School of Public Health, Harvard University, Boston, MA, United States.

^*^Corresponding author: [mary.labbe@utoronto.ca](mailto:mary.labbe@utoronto.ca)

Contents

[**Table 1.** Sample Characteristics, adults (19+ years old) 2](#_Toc126341438)

[**Table 2.** Comparison of calorie and nutrient composition between FID foods (n=239) and aggregated FLIP foods values, by food category (n=1481) 3](#_Toc126341439)

[**Table 3.** Baseline and counterfactual scenarios: Canadian adults’ (≥19 y) calorie and nutrient intakes, by food substitution counterfactual scenario tested (n=11,992) 7](#_Toc126341440)

[**Table 4.** Number of diet related NCD deaths that could be averted or delayed from substituting nutrient composition of packaged foods consumed by Canadian adults (≥19 y) with similar foods that would display at least one less ‘high in’ FOPL symbol - by cause of death (95% UI) 13](#_Toc126341441)

[**Table 5.** Age- and sex-specific estimates of the annual number of diet related NCD deaths in Canada, 2019 17](#_Toc126341442)

[**Table 6.** Age- and sex-specific estimates of the Canadian population, 2019 19](#_Toc126341443)

# **Table S1.** Sample Characteristics, adults (19+ years old)

| **Sex, n (%)** | **All (n=11,992)** |
| --- | --- |
| Males | 5,674 (50.1) |
| Females | 6,318 (49.9) |
| **Highest Level of Education**, n (%) |  |
| Less than high school diploma or its equivalent | 1,938 (11.8) |
| High school diploma or a high school  equivalency certificate | 3,066 (25.0) |
| Certificate/diploma -trade/college/non-university/university below Bachelor | 3,980 (34.2) |
| Bachelor’s degree or university certificate/diploma/degree above bachelor’s degree | 2,937 (28.5) |
| Not stated | 71 (0.6) |
| **Total Annual Household Income,** all sources n (%) |  |
| < $20,000 | 1,323 (7.9) |
| $20,000 – $39,999 | 2,520 (17.0) |
| $40,000 – $59,999 | 2,139 (16.5) |
| $60,000 – $79,999 | 1,679 (14.2) |
| $80,000 – $99,999 | 1,218 (11.1) |
| $100,000 – $119,999 | 992 (10.3) |
| $120,000 – $139,999 | 626 (6.9) |
| > $140,000 | 1,488 (16.0) |
| Not stated | 7 (0.1) |
| **Total household income,** main source n (%) |  |
| Employment income | 7709 (72.7) |
| Income from social benefits | 396 (2.4) |
| Senior’s benefits | 3128 (19.0) |
| Other | 422 (3.5) |
| Not stated | 337 (2.4) |
| **BMI**, n (%) |  |
| Normal weight (18.5 – 24.9 kg/m^2^) | 3,920 (32.7) |
| Overweight (25.0 – 29.9 kg/m^2^) | 4,409 (36.8) |
| Obese (>30.0 kg/m^2^) | 3,663 (30.6) |
| **Mean BMI**, by DRI age-sex category (CI) |  |
| 19 to 30 (m) | 26.14 (25.12, 27.16) |
| 19 to 30 (f) | 25.47 (24.67, 26.26) |
| 31 to 50 (m) | 28.50 (28.00, 28.98) |
| 31 to 50 (f) | 26.98 (26.52, 27.44) |
| 51 to 70 (m) | 29.05 (28.32, 29.79) |
| 51 to 70 (f) | 27.94 (27.33, 28.55) |
| 71+ (m) | 28.05 (27.53, 28.56) |
| 71+ (f) | 27.80 (27.03, 28.57) |
| **Height (m),** by DRI age-sex category (CI) |  |
| 19 to 30 (m) | 1.77 (1.76, 1.77) |
| 19 to 30 (f) | 1.64 (1.63, 1.65) |
| 31 to 50 (m) | 1.76 (1.76, 1.77) |
| 31 to 50 (f) | 1.63 (1.62, 1.64) |
| 51 to 70 (m) | 1.74 (1.73, 1.75) |
| 51 to 70 (f) | 1.61 (1.60, 1.62) |
| 71+ (m) | 1.72 (1.71, 1.73) |
| 71+ (f) | 1.58 (1.57, 1.58) |
| **Misreported**, n (%) |  |
| Under-reporter | 4,567 (37.9) |
| Plausible | 6,598 (55.3) |
| Over-reporter | 827 (6.8) |

# **Table S2.** Comparison of calorie and nutrient composition between FID foods (n=239) and aggregated FLIP foods values, by food category (n=1481)

| **TRA Food Category ^a^** | **Number of FID**  **food profiles (n)** | **Nutrient** | **FID food profiles** | | | | **Aggregated FLIP food profiles** | | | | **Absolute mean difference** | **p-value ^b^** |
| --- | --- | --- | --- | --- | --- | --- | --- | --- | --- | --- | --- | --- |
|  |  |  | **Mean (SD)** | **Min** | **50th (25th, 75th)** | **Max** | **Mean (SD)** | **Min** | **50th (25th, 75th)** | **Max** |  |  |
| **Bakery Products** | **49** | **Calories (kcal)** | **356.6 (97.2)** | 201 | 319 (278.0, 460.0) | 514 | **335.9 (98)** | 200 | 291.8 (255, 438.9) | 526.3 | -20.7 | 0.1275 |
|  |  | **Saturated Fat (g)** | **4 (4.6)** | 0 | 2.6 (0.7, 5.8) | 20.1 | **3.3 (4.1)** | 0.3 | 2.4 (0.6, 4.6) | 20 | -0.7 | 0.4303 |
|  |  | **Sodium (mg)** | **496.6 (184.3)** | 103 | 520 (353, 580) | 1002 | **360.2 (141.5)** | 5.1 | 359.4 (297.2, 399.2) | 736.8 | -136.4 | 0.0003* |
|  |  | **Sugars (g)** | **15.4 (15.5)** | 0 | 6.1 (3.9, 29) | 49.6 | **11.2 (11.5)** | 0 | 4.5 (2.3, 20) | 36.8 | -4.2 | 0.0478* |
|  |  | **Fibre (g)** | **4.1 (2.7)** | 1 | 3.3 (2, 6.2) | 12.6 | **4.5 (2.5)** | 0 | 4.2 (2.9, 5.8) | 10.8 | 0.4 | 0.1669 |
| **Beverages** | **8** | **Calories (kcal)** | **132.1 (184)** | 26 | 34 (32.8, 129.5) | 460 | **109.3 (190.1)** | 0 | 8.5 (5.8, 110.5) | 432.7 | -22.8 | 0.0657 |
|  |  | **Saturated Fat (g)** | **0.9 (1.8)** | 0 | 0 (0, 0.6) | 4.9 | **1.8 (3.7)** | 0 | 0 (0, 0.9) | 10.4 | 0.9 | 0.8481 |
|  |  | **Sodium (mg)** | **118 (187.8)** | 7 | 26.5 (11.3, 110.8) | 504 | **48.2 (58.7)** | 0 | 30.7 (4.7, 61) | 161.4 | -69.8 | 0.6454 |
|  |  | **Sugars (g)** | **21.1 (25.3)** | 5.2 | 8.8 (6.7, 21.3) | 65.6 | **16.6 (28.1)** | 0 | 1.9 (0.7, 17.6) | 63.7 | -4.5 | 0.0659 |
|  |  | **Fibre (g)** | **0.7 (1.4)** | 0 | 0 (0, 0.5) | 3.7 | **1.6 (3.1)** | 0 | 0 (0, 1.3) | 7.8 | 0.9 | 0.8360 |
| **Cereals and Other Grain Products** | **8** | **Calories (kcal)** | **420.9 (37.3)** | 386 | 404.5 (390.5, 459.2) | 475 | **370.4 (43.4)** | 285.3 | 371.8 (360.7, 386.3) | 440.6 | -50.5 | 0.0100* |
|  |  | **Saturated Fat (g)** | **2.9 (3.6)** | 0.3 | 1.3 (1, 3.1) | 10.8 | **0.7 (0.8)** | 0.2 | 0.4 (0.3, 0.6) | 2.5 | -2.2 | 0.0379* |
|  |  | **Sodium (mg)** | **605.5 (482.1)** | 7 | 538.5 (392, 751.8) | 1405 | **267.8 (168.7)** | 6.7 | 282.8 (140.9, 408.3) | 470.1 | -337.7 | 0.0650 |
|  |  | **Sugars (g)** | **25.2 (12.7)** | 5.7 | 28.3 (18.5, 35.5) | 37.3 | **19.3 (11.3)** | 0.9 | 23.6 (16.1, 26) | 29.2 | -5.9 | 0.2786 |
|  |  | **Fibre (g)** | **5.8 (1.6)** | 3.2 | 6.1 (5, 6.9) | 8 | **7 (2.7)** | 1.4 | 7.2 (6.4, 8.5) | 10 | 1.2 | 0.1889 |
| **Dairy Products and Substitutes** | **29** | **Calories (kcal)** | **156.6 (107)** | 33 | 99 (81, 257) | 420 | **153.9 (104.6)** | 17 | 93.7 (87.6, 233.3) | 403.9 | -2.7 | 0.9752 |
|  |  | **Saturated Fat (g)** | **5.9 (6.5)** | 0 | 1.9 (1.2, 9.1) | 19.3 | **5.8 (6.4)** | 0 | 2.4 (1, 11.1) | 18 | -0.1 | 0.7676 |
|  |  | **Sodium (mg)** | **343.3 (518.4)** | 8 | 52 (40, 406) | 1951 | **254.6 (349)** | 25 | 52.1 (42.9, 435.7) | 1450.7 | -88.7 | 0.9380 |
|  |  | **Sugars (g)** | **7.5 (5.1)** | 0 | 8 (3.2, 11.6) | 20.9 | **6.4 (4.2)** | 0 | 6 (3.2, 10.7) | 12.9 | -1.1 | 0.3794 |
|  |  | **Fibre (g)** | **0.1 (.)** | 0 | 0 (0, 0) | 1 | **0.2 (0.3)** | 0 | 0 (0, 0.3) | 1 | 0.1 | 0.0645 |
| **Desserts** | **13** | **Calories (kcal)** | **189.2 (111.9)** | 62 | 130 (127, 237) | 381 | **156.8 (136.8)** | 5.6 | 107.1 (77.3, 137.3) | 392.9 | -32.4 | 0.2377 |
|  |  | **Saturated Fat (g)** | **1.1 (0.8)** | 0 | 1 (0.5, 1.3) | 2.3 | **0.8 (0.6)** | 0 | 1 (0, 1.2) | 1.7 | -0.3 | 0.5024 |
|  |  | **Sodium (mg)** | **202.5 (191.1)** | 63 | 129 (92, 152) | 635 | **103.6 (78.5)** | 37 | 58.7 (52.1, 119.5) | 267.9 | -98.9 | 0.0455* |
|  |  | **Sugars (g)** | **29.4 (24.9)** | 11.6 | 19.2 (17, 22.1) | 86 | **24.1 (31.1)** | 0 | 11.5 (4.4, 18.3) | 88.4 | -5.3 | 0.0402* |
|  |  | **Fibre (g)** | **1.2 (2.1)** | 0 | 0 (0, 1.7) | 6.6 | **0.7 (1.1)** | 0 | 0.1 (0, 0.8) | 3.6 | -0.5 | 1.0000 |
| **Fats and Oils** | **19** | **Calories (kcal)** | **307.5 (148.9)** | 47 | 329 (200, 435) | 542 | **253.1 (111.6)** | 50.1 | 249.5 (155.8, 353.5) | 390.2 | -54.4 | 0.1290 |
|  |  | **Saturated Fat (g)** | **4.1 (3)** | 0.3 | 3 (1.3, 6.1) | 9.1 | **2.3 (1.4)** | 0 | 2.6 (1.4, 3.4) | 4.3 | -1.8 | 0.1020 |
|  |  | **Sodium (mg)** | **942.1 (302.2)** | 1 | 939 (852.5, 1097) | 1600 | **635.4 (167.9)** | 161.3 | 685 (622.4, 714.3) | 935.7 | -306.7 | 0.0000 |
|  |  | **Sugars (g)** | **10.7 (9.9)** | 1.9 | 6.7 (3.7, 15.6) | 38.7 | **9.3 (5.4)** | 0 | 9.7 (5.9, 12.6) | 18 | -1.4 | 0.6827 |
|  |  | **Fibre (g)** | **0.4 (0.5)** | 0 | 0.3 (0, 0.7) | 1.5 | **0 (0)** | 0 | 0 (0, 0) | 0 | -0.4 | 0.0000 |
| **Marine and Freshwater Animals** | **5** | **Calories (kcal)** | **163.6 (86.4)** | 95 | 117 (99, 217) | 290 | **153.7 (43.9)** | 100 | 173.9 (113.2, 187.5) | 194.1 | -9.9 | 1.0000 |
|  |  | **Saturated Fat (g)** | **1.4 (1.3)** | 0.2 | 0.9 (0.2, 2.8) | 2.8 | **1 (1.3)** | 0 | 0.5 (0, 1.6) | 3 | -0.4 | 0.6752 |
|  |  | **Sodium (mg)** | **695.8 (193.9)** | 490 | 672 (529, 870) | 918 | **366.7 (19.4)** | 340 | 373.9 (354.2, 377.4) | 388.2 | -329.1 | 0.0079* |
|  |  | **Sugars (g)** | **1.4 (2.7)** | 0 | 0 (0, 0.8) | 6.3 | **0.8 (1.8)** | 0 | 0 (0, 0) | 4 | -0.6 | 0.6072 |
|  |  | **Fibre (g)** | **1.3 (2.7)** | 0 | 0 (0, 0.5) | 6.1 | **0.6 (1.4)** | 0 | 0 (0, 0) | 3.1 | -0.7 | 0.6072 |
| **Fruit and Fruit Juices** | **13** | **Calories (kcal)** | **93.9 (97.9)** | 44 | 52 (46, 60) | 317 | **69.4 (100.9)** | 11 | 29.7 (20, 42.7) | 333.3 | -24.5 | 0.0088* |
|  |  | **Saturated Fat (g)** | **0 (0.1)** | 0 | 0 (0, 0) | 0.2 | **0 (0)** | 0 | 0 (0, 0) | 0 | 0 | 0.0000 |
|  |  | **Sodium (mg)** | **3.7 (1.9)** | 1 | 4 (2, 4.2) | 8 | **5.8 (6.2)** | 0 | 6.4 (0, 8) | 19.7 | 2.1 | 0.5878 |
|  |  | **Sugars (g)** | **20.5 (20.6)** | 8.3 | 11.9 (10.3, 14.5) | 67.5 | **9.7 (11.8)** | 2.2 | 4.8 (3.9, 8) | 36.7 | -10.8 | 0.0018* |
|  |  | **Fibre (g)** | **1.6 (2.3)** | 0 | 0.9 (0.2, 1.6) | 7.5 | **3.6 (7.9)** | 0 | 0.2 (0, 1.6) | 25 | 2 | 0.2882 |
| **Legumes** ^c^ | **1** | **Calories (kcal)** | **113 (.)** | 113 | 113 (113, 113) | 113 | **77.7 (.)** | 77.7 | 77.7 (77.7, 77.7) | 77.7 | -35.3 | 1.0000 |
|  |  | **Saturated Fat (g)** | **0.1 (.)** | 0.1 | 0.1 (0.1, 0.1) | 0.1 | **0 (.)** | 0 | 0 (0, 0) | 0 | -0.1 | 1.0000 |
|  |  | **Sodium (mg)** | **448 (.)** | 448 | 448 (448, 448) | 448 | **50.6 (.)** | 50.6 | 50.6 (50.6, 50.6) | 50.6 | -397.4 | 1.0000 |
|  |  | **Sugars (g)** | **0.3 (.)** | 0.3 | 0.3 (0.3, 0.3) | 0.3 | **0.2 (.)** | 0.2 | 0.2 (0.2, 0.2) | 0.2 | -0.1 | 1.0000 |
|  |  | **Fibre (g)** | **5.1 (.)** | 5.1 | 5.1 (5.1, 5.1) | 5.1 | **4.2 (.)** | 4.2 | 4.2 (4.2, 4.2) | 4.2 | -0.9 | 1.0000 |
| **Meats and Substitutes** | **27** | **Calories (kcal)** | **284 (101.4)** | 115 | 286 (240.5, 299.5) | 541 | **219 (91.8)** | 109.1 | 200 (154.1, 250.5) | 428.6 | -65 | 0.0056* |
|  |  | **Saturated Fat (g)** | **6.6 (3.8)** | 0.8 | 7 (3.9, 8) | 14.6 | **4.3 (3.9)** | 0 | 3 (1.1, 6.3) | 13.4 | -2.3 | 0.0134* |
|  |  | **Sodium (mg)** | **959.4 (374.5)** | 457 | 888 (704, 1123) | 2021 | **698.2 (309.2)** | 358.9 | 618.2 (509.2, 735) | 1733.3 | -261.2 | 0.0013* |
|  |  | **Sugars (g)** | **1.1 (1.1)** | 0 | 1.1 (0.4, 1.3) | 4.2 | **1.1 (1.3)** | 0 | 0.6 (0, 1.8) | 4.7 | 0 | 0.4847 |
|  |  | **Fibre (g)** | **0.5 (1.1)** | 0 | 0 (0, 0) | 4.9 | **0.5 (0.9)** | 0 | 0 (0, 0.7) | 3.9 | 0 | 0.5823 |
| **Combination Dishes** | **6** | **Calories (kcal)** | **170.3 (113.2)** | 90 | 103 (94, 258.2) | 325 | **131.9 (81.8)** | 87.5 | 99.1 (93.2, 112.3) | 297.7 | -38.4 | 0.6884 |
|  |  | **Saturated Fat (g)** | **2.3 (2.6)** | 0.1 | 1.5 (0.4, 3.3) | 6.8 | **1.2 (1.6)** | 0.1 | 0.4 (0.2, 1.4) | 4.2 | -1.1 | 0.5887 |
|  |  | **Sodium (mg)** | **489.8 (192.1)** | 343 | 421.5 (379, 500.8) | 861 | **297.4 (110.8)** | 200.3 | 252.3 (214.4, 375.7) | 461.1 | -192.4 | 0.0649 |
|  |  | **Sugars (g)** | **3.1 (3)** | 0.5 | 1.8 (1, 4.8) | 8 | **3.2 (2.1)** | 0.8 | 3 (1.6, 5) | 5.6 | 0.1 | 0.8182 |
|  |  | **Fibre (g)** | **3.5 (1.4)** | 1.9 | 3.7 (2.4, 4) | 5.5 | **3 (1.4)** | 0.9 | 3.4 (2.1, 4.1) | 4.5 | -0.5 | 0.8182 |
| **Sauces, Dips, Gravies and Condiments** | **6** | **Calories (kcal)** | **88.5 (53.9)** | 29 | 77.5 (51.8, 116.8) | 174 | **83.9 (32.2)** | 42.8 | 90.3 (57.2, 106.8) | 121.3 | -4.6 | 0.9372 |
|  |  | **Saturated Fat (g)** | **1 (2.4)** | 0 | 0 (0, 0.1) | 6 | **0.4 (0.8)** | 0 | 0 (0, 0.2) | 2 | -0.6 | 0.8033 |
|  |  | **Sodium (mg)** | **1900.3 (2444.7)** | 419 | 905.5 (735.8, 1481) | 6820 | **706.3 (446)** | 217.2 | 615 (446, 847.8) | 1475.4 | -1194 | 0.1797 |
|  |  | **Sugars (g)** | **5.5 (4.6)** | 0.4 | 4.7 (2, 8.7) | 11.8 | **7.5 (7.6)** | 0 | 4.8 (2.4, 13.1) | 18 | 2 | 0.8182 |
|  |  | **Fibre (g)** | **1.3 (0.6)** | 0.5 | 1.7 (0.8, 1.8) | 1.8 | **0.8 (0.7)** | 0 | 1 (0.2, 1.4) | 1.6 | -0.5 | 0.1689 |
| **Snacks** | **7** | **Calories (kcal)** | **545.9 (22.5)** | 519 | 545 (528.5, 558.5) | 583 | **515.9 (66.6)** | 390.2 | 530 (500.3, 548.7) | 593.3 | -30 | 0.5649 |
|  |  | **Saturated Fat (g)** | **10.3 (8.3)** | 5.4 | 7.2 (6.6, 8.5) | 29 | **7.1 (9.3)** | 1.2 | 3.3 (2.5, 6.3) | 27.6 | -3.2 | 0.0973 |
|  |  | **Sodium (mg)** | **554.6 (358.3)** | 6 | 679 (301, 826.5) | 942 | **387.4 (260.5)** | 3.7 | 365.9 (222.5, 581.3) | 734.3 | -167.2 | 0.2593 |
|  |  | **Sugars (g)** | **6 (13)** | 0.5 | 0.8 (0.7, 2.1) | 35.3 | **4 (5.4)** | 0 | 3.3 (0.6, 3.9) | 15.7 | -2 | 0.6200 |
|  |  | **Fibre (g)** | **6.2 (3.5)** | 1.4 | 7.7 (3.2, 9) | 10 | **6 (3.4)** | 0.7 | 5.2 (4.5, 8.5) | 10.3 | -0.2 | 1.0000 |
| **Soups** | **26** | **Calories (kcal)** | **124.5 (145.6)** | 4 | 64 (34.5, 154) | 446 | **113.6 (121.6)** | 3.8 | 78.4 (18.6, 180.6) | 358.5 | -10.9 | 0.7627 |
|  |  | **Saturated Fat (g)** | **1.6 (2)** | 0 | 1 (0.1, 2) | 8.1 | **0.7 (1.1)** | 0 | 0.2 (0, 1.1) | 4.8 | -0.9 | 0.0193* |
|  |  | **Sodium (mg)** | **3500.8 (7868.6)** | 143 | 300.5 (235.2, 986.8) | 26000 | **2174.3 (4509.5)** | 15.2 | 114.8 (71.1, 1148.9) | 16678.9 | -1326.5 | 0.0119* |
|  |  | **Sugars (g)** | **4.1 (6.1)** | 0.2 | 1.3 (0.4, 2.8) | 17.5 | **2.5 (4.1)** | 0 | 0.8 (0.4, 1.9) | 14.3 | -1.6 | 0.2374 |
|  |  | **Fibre (g)** | **0.9 (1.1)** | 0 | 0.6 (0.1, 1.2) | 3.2 | **0.9 (1.4)** | 0 | 0.2 (0, 1.4) | 4.8 | 0 | 0.1906 |
| **Sugars and Sweets** | **11** | **Calories (kcal)** | **487.5 (65.1)** | 348 | 516 (478.5, 527) | 550 | **481.4 (90.8)** | 292.2 | 517.1 (425.9, 544.3) | 598.5 | -6.1 | 0.7426 |
|  |  | **Saturated Fat (g)** | **13.5 (7.5)** | 0.3 | 14.6 (9.9, 18.8) | 24.1 | **16.9 (9)** | 0 | 21.6 (12.7, 23.7) | 25.7 | 3.4 | 0.2169 |
|  |  | **Sodium (mg)** | **135.3 (94.5)** | 23 | 89 (64.5, 223.5) | 290 | **150.8 (107.5)** | 33.5 | 106.8 (62.7, 227.9) | 344.4 | 15.5 | 0.7426 |
|  |  | **Sugars (g)** | **50.5 (8.6)** | 37.6 | 48.1 (46.3, 51.1) | 67.1 | **25.2 (13.8)** | 2.9 | 23.9 (17, 32.8) | 53.3 | -25.3 | 0.0003* |
|  |  | **Fibre (g)** | **2.7 (2.2)** | 0 | 3.2 (0.7, 3.8) | 7.2 | **5.3 (4.1)** | 0 | 4.5 (2.1, 8.8) | 11.3 | 2.6 | 0.1674 |
| **Vegetables** | **11** | **Calories (kcal)** | **45.7 (40.2)** | 12 | 25 (19, 57) | 146 | **57.7 (50.5)** | 17.7 | 28.6 (21.4, 73.9) | 153.9 | 12 | 0.4306 |
|  |  | **Saturated Fat (g)** | **0.2 (0.6)** | 0 | 0 (0, 0.1) | 2 | **0.3 (0.6)** | 0 | 0 (0, 0) | 1.9 | 0.1 | 0.0144* |
|  |  | **Sodium (mg)** | **739.6 (526.4)** | 169 | 661 (313, 991) | 1674 | **460.8 (331.9)** | 101.2 | 343.8 (203.1, 671.4) | 1198.5 | -278.8 | 0.1713 |
|  |  | **Sugars (g)** | **4.3 (4.4)** | 0.5 | 2.9 (1.9, 4.1) | 13.6 | **2.9 (3.6)** | 0 | 2.1 (0.1, 3.6) | 10.4 | -1.4 | 0.2367 |
|  |  | **Fibre (g)** | **2 (1.2)** | 0.4 | 1.9 (0.9, 2.9) | 4.1 | **2 (.)** | 0.2 | 2.2 (0.9, 3.3) | 3.8 | 0 | 0.9177 |

^a^ Major categories were defined as per Health Canada’s Table of Reference Amounts for Foods (1).

^b^ P-value determined using Mann Whitney U tests. *Indicates a statistically significant difference.

^c^ Unable to determine SD for the category “Legumes” because there was only one food profile in this category.

Abbreviations: FID = Food and Ingredient Details; FLIP = Food Label Information and Price; Kcal = calories; g = grams; mg = milligrams; SD = Standard Deviation.

# **Table S3.** Baseline and counterfactual scenarios: Canadian adults’ (≥19 y) calorie and nutrient intakes, by food substitution counterfactual scenario tested (n=11,992)

| **Table S3.1.** Potential changes in usual energy intake from substituting nutrient composition of packaged foods consumed by Canadian adults (≥19 y) with similar foods that would display at least one less ‘high in’ FOPL symbol, overall and by DRI age-sex group. | | | | | | | | | | | | | | | |
| --- | --- | --- | --- | --- | --- | --- | --- | --- | --- | --- | --- | --- | --- | --- | --- |
| ***ENERGY (kcal/d)*** | | | | | | | | | | | | | | | |
|  | ***n*** | ***Baseline mean*** | *SE* | ***S1 mean*** | *SE* | *S1 ∆* | ***S2 mean*** | *SE* | *S2 ∆* | ***S3 mean*** | *SE* | *S3 ∆* | ***S4 mean*** | *SE* | *S4 ∆* |
| ***Total 19+*** | ***11991*** | ***1889*** | *20* | ***1875*** | *21* | ***14*** | ***1865*** | *20* | ***23*** | ***1857*** | *21* | ***32*** | ***1842*** | *21* | ***46*** |
| ***Males*** | | | | | | | | | | | | | | | |
| 19-30 y | 765 | **2373** | 51 | **2355** | 50 | **18** | **2341** | 50 | **32** | **2332** | 50 | **41** | **2314** | 49 | **59** |
| 31-50 y | 1839 | **2236** | 36 | **2219** | 36 | **17** | **2206** | 36 | **30** | **2198** | 36 | **38** | **2181** | 36 | **55** |
| 51-70 y | 1964 | **2039** | 54 | **2024** | 55 | **15** | **2013** | 54 | **26** | **2005** | 55 | **35** | **1989** | 54 | **50** |
| > 70 y | 1105 | **1905** | 88 | **1892** | 89 | **14** | **1881** | 88 | **24** | **1873** | 88 | **32** | **1859** | 87 | **47** |
| ***Females*** | | | | | | | | | | | | | | | |
| 19-30 y | 815 | **1710** | 78 | **1698** | 77 | **13** | **1690** | 77 | **20** | **1681** | 77 | **29** | **1669** | 75 | **42** |
| 31-50 y | 2056 | **1665** | 32 | **1653** | 32 | **12** | **1646** | 31 | **19** | **1636** | 31 | **28** | **1624** | 30 | **40** |
| 51-70 y | 2107 | **1585** | 25 | **1574** | 25 | **11** | **1568** | 25 | **18** | **1559** | 25 | **26** | **1547** | 25 | **38** |
| > 70 y | 1340 | **1464** | 36 | **1454** | 36 | **10** | **1449** | 36 | **16** | **1440** | 36 | **24** | **1429** | 35 | **35** |

Baseline and counterfactual calorie and nutrient intakes were estimated using *CCHS-Nutrition 2015 PUMF* data (2, 3). Usual intakes were estimated using the National Cancer Institute (NCI) method, and analyses were adjusted for age, sex, dietary misreporting status, weekend/weekday, and sequence of dietary recall. Baseline and counterfactual scenarios are described in the methods section. Food substitution was performed for 30% (S1), 50% (S2), and 70% (S3) of randomly selected *CCHS-Nutrition 2015* adult participants and for all adult participants (S4) who consumed at least one packaged food product that would display a ‘high in' FOPL symbol under Canadian FOPL regulations. Abbreviations: d=day; g=grams; SE=standard error; FOPL= front-of-pack labeling; S1=scenario 1; S2=scenario 2; S3=scenario 3; S4=scenario 4.

| **Table S3.2.** Potential changes in usual sodium intake from substituting nutrient composition of packaged foods consumed by Canadian adults (≥19 y) with similar foods that would display at least one less ‘high in’ FOPL symbol, overall and by DRI age-sex group. | | | | | | | | | | | | | | | |
| --- | --- | --- | --- | --- | --- | --- | --- | --- | --- | --- | --- | --- | --- | --- | --- |
| ***SODIUM (mg/d)*** | | | | | | | | | | | | | | | |
|  | ***n*** | ***Baseline mean*** | *SE* | ***S1 mean*** | *SE* | *S1 ∆* | ***S2 mean*** | *SE* | *S2 ∆* | ***S3 mean*** | *SE* | *S3 ∆* | ***S4 mean*** | *SE* | *S4 ∆* |
| ***Total 19+*** | ***11992*** | ***2729*** | *33* | ***2656*** | *37* | ***73*** | ***2598**** | *32* | ***131*** | ***2548**** | *30* | ***182*** | ***2470**** | *31* | ***259*** |
| ***Males*** | | | | | | | | | | | | | | | |
| 19-30 y | 765 | **3435** | 85 | **3353** | 83 | **82** | **3282** | 83 | **153** | **3220** | 82 | **214** | **3145** | 80 | **290** |
| 31-50 y | 1839 | **3247** | 66 | **3163** | 67 | **84** | **3093** | 61 | **154** | **3033** | 60 | **213** | **2950*** | 59 | **297** |
| 51-70 y | 1965 | **2974** | 86 | **2889** | 92 | **85** | **2822** | 82 | **152** | **2765** | 78 | **209** | **2674** | 76 | **300** |
| > 70 y | 1105 | **2775** | 126 | **2689** | 132 | **87** | **2623** | 123 | **152** | **2569** | 119 | **206** | **2472** | 113 | **303** |
| ***Females*** | | | | | | | | | | | | | | | |
| 19-30 y | 815 | **2448** | 122 | **2391** | 114 | **58** | **2342** | 119 | **107** | **2299** | 118 | **150** | **2242** | 108 | **207** |
| 31-50 y | 2056 | **2387** | 52 | **2326** | 46 | **61** | **2278** | 48 | **110** | **2235** | 48 | **153** | **2169*** | 43 | **218** |
| 51-70 y | 2107 | **2272** | 31 | **2207** | 32 | **65** | **2160** | 32 | **111** | **2118*** | 30 | **154** | **2045*** | 30 | **227** |
| > 70 y | 1340 | **2107** | 45 | **2041** | 48 | **66** | **1996** | 49 | **111** | **1956** | 46 | **151** | **1877*** | 45 | **230** |

Baseline and counterfactual calorie and nutrient intakes were estimated using *CCHS-Nutrition 2015 PUMF* data (2, 3). Usual intakes were estimated using the National Cancer Institute (NCI) method, and analyses were adjusted for age, sex, dietary misreporting status, weekend/weekday, and sequence of dietary recall. Baseline and counterfactual scenarios are described in the methods section. Food substitution was performed for 30% (S1), 50% (S2), and 70% (S3) of randomly selected *CCHS-Nutrition 2015* adult participants and for all adult participants (S4) who consumed at least one packaged food product that would display a ‘high in' FOPL symbol under Canadian FOPL regulations. *Indicates a statistically significant difference between baseline mean intakes and counterfactual mean intakes. Abbreviations: d=day; g=grams; SE=standard error; FOPL= front-of-pack labeling; S1=scenario 1; S2=scenario 2; S3=scenario 3; S4=scenario 4.

| **Table S3.3.** Potential changes in usual total sugar intake from substituting nutrient composition of packaged foods consumed by Canadian adults (≥19 y) with similar foods that would display at least one less ‘high in’ FOPL symbol, overall and by DRI age-sex group. | | | | | | | | | | | | | | | |
| --- | --- | --- | --- | --- | --- | --- | --- | --- | --- | --- | --- | --- | --- | --- | --- |
| ***SUGARS (g/d)*** | | | | | | | | | | | | | | | |
|  | ***n*** | ***Baseline mean*** | *SE* | ***S1 mean*** | *SE* | *S1 ∆* | ***S2 mean*** | *SE* | *S2 ∆* | ***S3 mean*** | *SE* | *S3 ∆* | ***S4 mean*** | *SE* | *S4 ∆* |
| ***Total 19+*** | ***11988*** | ***86.43*** | *0.95* | ***84.43*** | *0.91* | ***2.01*** | ***82.91*** | *0.91* | ***3.52*** | ***81.59**** | *0.90* | ***4.84*** | ***79.50**** | *0.88* | ***6.93*** |
| ***Males*** | | | | | | | | | | | | | | | |
| 19-30 y | 765 | **101.36** | 3.49 | **98.86** | 3.21 | **2.50** | **96.55** | 3.35 | **4.81** | **95.33** | 3.11 | **6.03** | **92.67** | 2.97 | **8.69** |
| 31-50 y | 1837 | **96.58** | 2.11 | **94.30** | 1.94 | **2.28** | **92.22** | 2.03 | **4.37** | **91.13** | 1.89 | **5.45** | **88.69*** | 1.81 | **7.89** |
| 51-70 y | 1964 | **90.31** | 2.38 | **88.31** | 2.54 | **2.01** | **86.49** | 2.29 | **3.82** | **85.57** | 2.39 | **4.74** | **83.41** | 2.36 | **6.90** |
| > 70 y | 1105 | **86.01** | 4.41 | **84.20** | 4.48 | **1.80** | **82.60** | 4.21 | **3.41** | **81.80** | 4.18 | **4.20** | **79.85** | 4.10 | **6.15** |
| ***Females*** | | | | | | | | | | | | | | | |
| 19-30 y | 815 | **81.02** | 5.02 | **78.97** | 4.83 | **2.05** | **77.73** | 4.74 | **3.30** | **75.92** | 4.59 | **5.11** | **73.91** | 4.43 | **7.11** |
| 31-50 y | 2056 | **80.14** | 2.21 | **78.22** | 2.14 | **1.93** | **77.09** | 2.06 | **3.05** | **75.40** | 2.06 | **4.75** | **73.48** | 2.01 | **6.66** |
| 51-70 y | 2107 | **77.88** | 1.20 | **76.12** | 1.17 | **1.76** | **75.15** | 1.21 | **2.72** | **73.61** | 1.12 | **4.27** | **71.83*** | 1.10 | **6.05** |
| > 70 y | 1339 | **73.86** | 2.32 | **72.30** | 2.24 | **1.56** | **71.52** | 2.29 | **2.34** | **70.13** | 2.06 | **3.73** | **68.54** | 1.99 | **5.31** |

Baseline and counterfactual calorie and nutrient intakes were estimated using *CCHS-Nutrition 2015 PUMF* data (2, 3). Usual intakes were estimated using the National Cancer Institute (NCI) method, and analyses were adjusted for age, sex, dietary misreporting status, weekend/weekday, and sequence of dietary recall. Baseline and counterfactual scenarios are described in the methods section. Food substitution was performed for 30% (S1), 50% (S2), and 70% (S3) of randomly selected *CCHS-Nutrition 2015* adult participants and for all adult participants (S4) who consumed at least one packaged food product that would display a ‘high in' FOPL symbol under Canadian FOPL regulations. *Indicates a statistically significant difference between baseline mean intakes and counterfactual mean intakes. Abbreviations: d=day; g=grams; SE=standard error; FOPL= front-of-pack labeling; S1=scenario 1; S2=scenario 2; S3=scenario 3; S4=scenario 4.

| **Table S3.4.** Potential changes in usual saturated fat intake from substituting nutrient composition of packaged foods consumed by Canadian adults (≥19 y) with similar foods that would display at least one less ‘high in’ FOPL symbol, overall and by DRI age-sex group. | | | | | | | | | | | | | | | |
| --- | --- | --- | --- | --- | --- | --- | --- | --- | --- | --- | --- | --- | --- | --- | --- |
| ***SATURATED FAT (g/d)*** | | | | | | | | | | | | | | | |
|  | ***n*** | ***Baseline mean*** | *SE* | ***S1 mean*** | *SE* | *S1 ∆* | ***S2 mean*** | *SE* | *S2 ∆* | ***S3 mean*** | *SE* | *S3 ∆* | ***S4 mean*** | *SE* | *S4 ∆* |
| ***Total 19+*** | ***11991*** | ***22.84*** | *0.56* | ***22.69*** | *0.56* | ***0.15*** | ***22.60*** | *0.57* | ***0.24*** | ***22.52*** | *0.57* | ***0.32*** | ***22.36*** | *0.58* | ***0.48*** |
| ***Males*** | | | | | | | | | | | | | | | |
| 19-30 y | 765 | **28.87** | 0.82 | **28.66** | 0.81 | **0.21** | **28.50** | 0.81 | **0.37** | **28.36** | 0.81 | **0.51** | **28.16** | 0.81 | **0.71** |
| 31-50 y | 1839 | **27.01** | 0.71 | **26.83** | 0.71 | **0.18** | **26.69** | 0.70 | **0.32** | **26.57** | 0.70 | **0.44** | **26.37** | 0.70 | **0.64** |
| 51-70 y | 1964 | **24.39** | 1.13 | **24.26** | 1.13 | **0.13** | **24.14** | 1.13 | **0.25** | **24.04** | 1.15 | **0.35** | **23.86** | 1.13 | **0.53** |
| > 70 y | 1105 | **22.56** | 1.60 | **22.46** | 1.59 | **0.10** | **22.36** | 1.61 | **0.20** | **22.28** | 1.63 | **0.28** | **22.11** | 1.60 | **0.45** |
| ***Females*** | | | | | | | | | | | | | | | |
| 19-30 y | 815 | **20.83** | 1.00 | **20.66** | 1.00 | **0.17** | **20.59** | 0.98 | **0.24** | **20.51** | 0.98 | **0.32** | **20.39** | 0.95 | **0.44** |
| 31-50 y | 2056 | **20.28** | 0.38 | **20.14** | 0.38 | **0.14** | **20.07** | 0.38 | **0.21** | **20.01** | 0.38 | **0.27** | **19.89** | 0.38 | **0.39** |
| 51-70 y | 2107 | **19.30** | 0.61 | **19.18** | 0.60 | **0.12** | **19.12** | 0.62 | **0.18** | **19.08** | 0.63 | **0.22** | **18.95** | 0.64 | **0.35** |
| > 70 y | 1340 | **17.74** | 0.80 | **17.65** | 0.79 | **0.09** | **17.61** | 0.82 | **0.13** | **17.58** | 0.83 | **0.16** | **17.45** | 0.83 | **0.29** |

Baseline and counterfactual calorie and nutrient intakes were estimated using *CCHS-Nutrition 2015 PUMF* data (2, 3). Usual intakes were estimated using the National Cancer Institute (NCI) method, and analyses were adjusted for age, sex, dietary misreporting status, weekend/weekday, and sequence of dietary recall. Baseline and counterfactual scenarios are described in the methods section. Food substitution was performed for 30% (S1), 50% (S2), and 70% (S3) of randomly selected *CCHS-Nutrition 2015* adult participants and for all adult participants (S4) who consumed at least one packaged food product that would display a ‘high in' FOPL symbol under Canadian FOPL regulations. Abbreviations: d=day; g=grams; SE=standard error; FOPL= front-of-pack labeling; S1=scenario 1; S2=scenario 2; S3=scenario 3; S4=scenario 4.

| **Table S3.5.** Potential changes in percentage of total energy from saturated fat intake from substituting nutrient composition of packaged foods consumed by Canadian adults (≥19 y) with similar foods that would display at least one less ‘high in’ FOPL symbol, overall and by DRI age-sex group. | | | | | | | | | | | | | | | |
| --- | --- | --- | --- | --- | --- | --- | --- | --- | --- | --- | --- | --- | --- | --- | --- |
| ***SATURATED FAT (% total energy/d)*** | | | | | | | | | | | | | | | |
|  | ***n*** | ***Baseline mean*** | *SE* | ***S1 mean*** | *SE* | *S1 ∆* | ***S2 mean*** | *SE* | *S2 ∆* | ***S3 mean*** | *SE* | *S3 ∆* | ***S4 mean*** | *SE* | *S4 ∆* |
| ***Total 19+*** | ***11991*** | ***10.56*** | *0.18* | ***10.57*** | *0.18* | ***-0.01*** | ***10.58*** | *0.18* | ***-0.02*** | ***10.59*** | *0.19* | ***-0.03*** | ***10.60*** | *0.19* | ***-0.04*** |
| ***Males*** | | | | | | | | | | | | | | | |
| 19-30 y | 765 | **10.73** | 0.15 | **10.73** | 0.15 | **0.00** | **10.72** | 0.15 | **0.01** | **10.71** | 0.15 | **0.02** | **10.72** | 0.15 | **0.01** |
| 31-50 y | 1839 | **10.60** | 0.15 | **10.61** | 0.14 | **-0.01** | **10.61** | 0.14 | **-0.01** | **10.60** | 0.14 | **0.00** | **10.61** | 0.14 | **-0.01** |
| 51-70 y | 1964 | **10.44** | 0.23 | **10.46** | 0.23 | **-0.02** | **10.47** | 0.24 | **-0.03** | **10.47** | 0.24 | **-0.03** | **10.48** | 0.24 | **-0.04** |
| > 70 y | 1105 | **10.31** | 0.31 | **10.34** | 0.30 | **-0.03** | **10.36** | 0.33 | **-0.05** | **10.37** | 0.33 | **-0.06** | **10.37** | 0.33 | **-0.06** |
| ***Females*** | | | | | | | | | | | | | | | |
| 19-30 y | 815 | **10.66** | 0.15 | **10.66** | 0.15 | **0.00** | **10.65** | 0.15 | **0.01** | **10.67** | 0.15 | **-0.01** | **10.69** | 0.15 | **-0.03** |
| 31-50 y | 2056 | **10.63** | 0.17 | **10.63** | 0.17 | **0.00** | **10.63** | 0.17 | **0.00** | **10.66** | 0.17 | **-0.02** | **10.67** | 0.18 | **-0.04** |
| 51-70 y | 2107 | **10.56** | 0.26 | **10.58** | 0.25 | **-0.01** | **10.59** | 0.27 | **-0.03** | **10.62** | 0.27 | **-0.06** | **10.63** | 0.28 | **-0.07** |
| > 70 y | 1340 | **10.43** | 0.32 | **10.46** | 0.31 | **-0.03** | **10.48** | 0.33 | **-0.05** | **10.52** | 0.34 | **-0.09** | **10.53** | 0.35 | **-0.10** |

Baseline and counterfactual calorie and nutrient intakes were estimated using *CCHS-Nutrition 2015 PUMF* data (2, 3). Usual intakes were estimated using the National Cancer Institute (NCI) method, and analyses were adjusted for age, sex, dietary misreporting status, weekend/weekday, and sequence of dietary recall. Baseline and counterfactual scenarios are described in the methods section. Food substitution was performed for 30% (S1), 50% (S2), and 70% (S3) of randomly selected *CCHS-Nutrition 2015* adult participants and for all adult participants (S4) who consumed at least one packaged food product that would display a ‘high in' FOPL symbol under Canadian FOPL regulations. Abbreviations: d=day; g=grams; SE=standard error; FOPL= front-of-pack labeling; S1=scenario 1; S2=scenario 2; S3=scenario 3; S4=scenario 4.

| **Table S3.6.** Potential changes in usual fiber intake from substituting nutrient composition of packaged foods consumed by Canadian adults (≥19 y) with similar foods that would display at least one less ‘high in’ FOPL symbol, overall and by DRI age-sex group. | | | | | | | | | | | | | | | |
| --- | --- | --- | --- | --- | --- | --- | --- | --- | --- | --- | --- | --- | --- | --- | --- |
| ***FIBER (g/d)*** | | | | | | | | | | | | | | | |
|  | ***n*** | ***Baseline mean*** | *SE* | ***S1 mean*** | *SE* | *S1 ∆* | ***S2 mean*** | *SE* | *S2 ∆* | ***S3 mean*** | *SE* | *S3 ∆* | ***S4 mean*** | *SE* | *S4 ∆* |
| ***Total 19+*** | ***11988*** | ***17.27*** | *0.22* | ***17.28*** | *0.21* | ***-0.01*** | ***17.27*** | *0.20* | ***0.00*** | ***17.25*** | *0.20* | ***0.01*** | ***17.24*** | *0.19* | ***0.02*** |
| ***Males*** | | | | | | | | | | | | | | | |
| 19-30 y | 765 | **18.44** | 0.44 | **18.51** | 0.43 | **-0.08** | **18.50** | 0.44 | **-0.06** | **18.52** | 0.45 | **-0.08** | **18.58** | 0.44 | **-0.14** |
| 31-50 y | 1839 | **18.39** | 0.38 | **18.42** | 0.37 | **-0.03** | **18.40** | 0.38 | **0.00** | **18.40** | 0.37 | **-0.01** | **18.42** | 0.36 | **-0.03** |
| 51-70 y | 1965 | **18.18** | 0.29 | **18.16** | 0.30 | **0.03** | **18.12** | 0.29 | **0.06** | **18.11** | 0.30 | **0.08** | **18.08** | 0.31 | **0.11** |
| > 70 y | 1105 | **18.22** | 0.44 | **18.14** | 0.45 | **0.08** | **18.10** | 0.44 | **0.12** | **18.07** | 0.47 | **0.15** | **17.99** | 0.47 | **0.23** |
| ***Females*** | | | | | | | | | | | | | | | |
| 19-30 y | 814 | **15.61** | 0.66 | **15.69** | 0.64 | **-0.08** | **15.71** | 0.61 | **-0.10** | **15.71** | 0.64 | **-0.10** | **15.75** | 0.62 | **-0.14** |
| 31-50 y | 2054 | **16.12** | 0.40 | **16.16** | 0.39 | **-0.04** | **16.17** | 0.37 | **-0.05** | **16.15** | 0.38 | **-0.03** | **16.15** | 0.37 | **-0.04** |
| 51-70 y | 2107 | **16.48** | 0.25 | **16.46** | 0.24 | **0.01** | **16.47** | 0.23 | **0.01** | **16.43** | 0.23 | **0.04** | **16.39** | 0.23 | **0.09** |
| > 70 y | 1339 | **16.54** | 0.35 | **16.47** | 0.35 | **0.07** | **16.47** | 0.34 | **0.07** | **16.42** | 0.33 | **0.12** | **16.33** | 0.33 | **0.21** |

Baseline and counterfactual calorie and nutrient intakes were estimated using *CCHS-Nutrition 2015 PUMF* data (2, 3). Usual intakes were estimated using the National Cancer Institute (NCI) method, and analyses were adjusted for age, sex, dietary misreporting status, weekend/weekday, and sequence of dietary recall. Baseline and counterfactual scenarios are described in the methods section. Food substitution was performed for 30% (S1), 50% (S2), and 70% (S3) of randomly selected *CCHS-Nutrition 2015* adult participants and for all adult participants (S4) who consumed at least one packaged food product that would display a ‘high in' FOPL symbol under Canadian FOPL regulations. Abbreviations: d=day; g=grams; SE=standard error; FOPL= front-of-pack labeling; S1=scenario 1; S2=scenario 2; S3=scenario 3; S4=scenario 4.

# **Table S4.** Number of diet related NCD deaths that could be averted or delayed from substituting nutrient composition of packaged foods consumed by Canadian adults (≥19 y) with similar foods that would display at least one less ‘high in’ FOPL symbol - by cause of death (95% UI)

| **Table S4.1.** Scenario 1: Food substitution for 30% of randomly selected *CCHS-Nutrition 2015* adult participants who consumed at least one packaged food product that would display a ‘high in' FOPL symbol | | | | | | |
| --- | --- | --- | --- | --- | --- | --- |
| **Cause of death (ICD-10 Code) ^1^** | **Total (mean, 95% UI) ^2^** | **% ^3^** | **Males (mean, 95% UI) ^2^** | **% ^3^** | **Females (mean, 95% UI) ^2^** | **% ^3^** |
| **Cardiovascular diseases** | **1499 (1284, 1716)** | **69.8** | **847 (722, 978)** | **70.4** | **655 (546, 769)** | **68.9** |
| Ischaemic heart disease (I20-25) | 739 (626, 852) | 34.4 | 494 (417, 575) | 41.1 | 244 (183, 306) | 25.7 |
| Cerebrovascular disease (I60-69) | 284 (216, 352) | 13.2 | 133 (102, 163) | 11.1 | 152 (117, 190) | 16.0 |
| Heart failure (I50) | 251 (172, 327) | 11.7 | 116 (79, 152) | 9.6 | 135 (91, 178) | 14.2 |
| Aortic aneurysm (I71) | 9 (4, 15) | 0.4 | 6 (2, 10) | 0.5 | 3 (1, 5) | 0.3 |
| Pulmonary embolism (I26) | 2 (1, 4) | 0.1 | 1 (0, 2) | 0.1 | 1 (0, 2) | 0.1 |
| Rheumatic heart disease (I05-09) | 2 (0, 3) | 0.1 | 1 (0, 1) | 0.1 | 1 (0, 2) | 0.1 |
| Hypertensive disease (I10-15) | 215 (165, 263) | 10.0 | 97 (74, 119) | 8.1 | 120 (93, 146) | 12.6 |
| **Diabetes (E11, E14)** | **293 (225, 353)** | **13.6** | **165 (129, 199)** | **13.7** | **127 (95, 155)** | **13.4** |
| **Cancer** | **187 (145, 229)** | **8.7** | **85 (63, 107)** | **7.1** | **101 (79, 124)** | **10.6** |
| Colorectum (C18-C20) | 83 (55, 112) | 3.9 | 44 (29, 60) | 3.7 | 38 (25, 51) | 4.0 |
| Gallbladder (C23) | 4 (2, 5) | 0.2 | 1 (1, 2) | 0.1 | 2 (1, 3) | 0.2 |
| Pancreas (C25) | 34 (7, 63) | 1.6 | 18 (3, 32) | 1.5 | 16 (3, 30) | 1.7 |
| Breast (C50) | 7 (-4, 18) | 0.3 | 0 | 0.0 | 7 (-4, 8) | 0.7 |
| Endometrium (C54.1) | 26 (19, 34) | 1.2 | 0 | 0.0 | 26 (19, 34) | 2.7 |
| Kidney (C64) | 33 (26, 40) | 1.5 | 21 (17, 26) | 1.7 | 11 (9, 14) | 1.2 |
| **Chronic renal failure (N18)** | **58 (29, 88)** | **2.7** | **30 (15, 45)** | **2.5** | **28 (13, 43)** | **2.9** |
| **Liver disease (K70, K74)** | **113 (70, 154)** | **5.3** | **76 (50, 103)** | **6.3** | **37 (21, 52)** | **3.9** |
| *Total deaths prevented under 75* | *782 (695, 866)* | *36.4* | *545 (483, 608)* | *45.3* | *237 (208, 265)* | *24.9* |
| **Total deaths averted or delayed** | **2148 (1913, 2386)** | **100.0** | **1203 (1064, 1343)** | **100.0** | **950 (832, 1068)** | **100.0** |
| *Actual number of diet-related NCD deaths in Canada (2019)* | *92845* | | *46568* | | *46277* | |
| ***% of actual diet-related NCD deaths that could be averted or delayed* ^4^** | ***2.3*** | | ***2.6*** | | ***2.1*** | |
| **Table S4.2.** Scenario 2: Food substitution for 50% of randomly selected *CCHS-Nutrition 2015* adult participants who consumed at least one packaged food product that would display a ‘high in' FOPL symbol | | | | | | |
| **Cause of death (ICD-10 Code) ^1^** | **Total (mean, 95% UI) ^2^** | **% ^3^** | **Males (mean, 95% UI) ^2^** | **% ^3^** | **Females (mean, 95% UI) ^2^** | **% ^3^** |
| **Cardiovascular diseases** | **2500 (2122, 2872)** | **70.2** | **1502 (1279, 1729)** | **70.5** | **1002 (823, 1176)** | **69.5** |
| Ischaemic heart disease (I20-25) | 1254 (1065, 1449) | 35.2 | 882 (743, 1017) | 41.4 | 377 (282, 471) | 26.2 |
| Cerebrovascular disease (I60-69) | 468 (353, 579) | 13.1 | 235 (180, 290) | 11.0 | 233 (176, 289) | 16.2 |
| Heart failure (I50) | 406 (279, 529) | 11.4 | 205 (143, 266) | 9.6 | 202 (134, 268) | 14.0 |
| Aortic aneurysm (I71) | 15 (6, 26) | 0.4 | 11 (4, 18) | 0.5 | 5 (2, 8) | 0.3 |
| Pulmonary embolism (I26) | 3 (1, 7) | 0.1 | 2 (1, 4) | 0.1 | 2 (0, 3) | 0.1 |
| Rheumatic heart disease (I05-09) | 3 (1, 6) | 0.1 | 1 (0, 2) | 0.0 | 2 (0, 3) | 0.1 |
| Hypertensive disease (I10-15) | 349 (266, 425) | 9.8 | 169 (131, 207) | 7.9 | 182 (137, 224) | 12.6 |
| **Diabetes (E11, E14)** | **480 (370, 579)** | **13.5** | **291 (227, 348)** | **13.7** | **190 (139, 230)** | **13.2** |
| **Cancer** | **308 (237, 377)** | **8.6** | **153 (113, 192)** | **7.2** | **154 (120, 188)** | **10.7** |
| Colorectum (C18-C20) | 140 (91, 187) | 3.9 | 80 (52, 109) | 3.8 | 59 (39, 79) | 4.1 |
| Gallbladder (C23) | 6 (4, 8) | 0.2 | 2 (2, 3) | 0.1 | 3 (2, 4) | 0.2 |
| Pancreas (C25) | 58 (11, 102) | 1.6 | 32 (6, 58) | 1.5 | 25 (4, 46) | 1.7 |
| Breast (C50) | 10 (-7, 26) | 0.3 | 0 | 0.0 | 10 (-6, 26) | 0.7 |
| Endometrium (C54.1) | 40 (28, 51) | 1.1 | 0 | 0.0 | 40 (29, 50) | 2.8 |
| Kidney (C64) | 56 (44, 67) | 1.6 | 38 (30, 46) | 1.8 | 17 (14, 21) | 1.2 |
| **Chronic renal failure (N18)** | **96 (45, 145)** | **2.7** | **53 (26, 81)** | **2.5** | **41 (19, 63)** | **2.8** |
| **Liver disease (K70, K74)** | **187 (117, 253)** | **5.2** | **132 (84, 177)** | **6.2** | **55 (30, 77)** | **3.8** |
| *Total deaths prevented under 75* | *1308 (1162, 1458)* | *36.7* | *946 (835, 1053)* | *44.4* | *365 (317, 411)* | *25.3* |
| **Total deaths averted or delayed** | **3563 (3163, 3969)** | **100.0** | **2129 (1886, 2372)** | **100.0** | **1441 (1247, 1625)** | **100.0** |
| *Actual number of diet-related NCD deaths in Canada (2019)* | *92845* | | *46568* | | *46277* | |
| ***% of actual diet-related NCD deaths that could be averted or delayed* ^4^** | ***3.8*** | | ***4.6*** | | ***3.1*** | |

| **Table S4.3.** Scenario 3: Food substitution for 70% of randomly selected *CCHS-Nutrition 2015* adult participants who consumed at least one packaged food product that would display a ‘high in' FOPL symbol | | | | | | |
| --- | --- | --- | --- | --- | --- | --- |
| **Cause of death (ICD-10 Code) ^1^** | **Total (mean, 95% UI) ^2^** | **% ^3^** | **Males (mean, 95% UI) ^2^** | **% ^3^** | **Females (mean, 95% UI) ^2^** | **% ^3^** |
| **Cardiovascular diseases** | **3482 (2984, 3994)** | **69.9** | **1982 (1687, 2283)** | **70.8** | **1503 (1259, 1757)** | **68.9** |
| Ischaemic heart disease (I20-25) | 1732 (1468, 1994) | 34.8 | 1168 (988, 1354) | 41.7 | 565 (420, 710) | 25.9 |
| Cerebrovascular disease (I60-69) | 660 (505, 813) | 13.3 | 309 (237, 382) | 11.0 | 349 (265, 437) | 16.0 |
| Heart failure (I50) | 576 (388, 753) | 11.6 | 269 (188, 348) | 9.6 | 308 (204, 409) | 14.1 |
| Aortic aneurysm (I71) | 21 (9, 35) | 0.4 | 14 (6, 24) | 0.5 | 7 (3, 11) | 0.3 |
| Pulmonary embolism (I26) | 4 (1, 9) | 0.1 | 2 (1, 5) | 0.1 | 2 (1, 4) | 0.1 |
| Rheumatic heart disease (I05-09) | 4 (1, 8) | 0.1 | 2 (0, 3) | 0.1 | 2 (1, 4) | 0.1 |
| Hypertensive disease (I10-15) | 490 (382, 598) | 9.8 | 221 (172, 271) | 7.9 | 271 (209, 328) | 12.4 |
| **Diabetes (E11, E14)** | **671 (514, 808)** | **13.5** | **377 (293, 455)** | **13.5** | **290 (216, 354)** | **13.3** |
| **Cancer** | **445 (343, 549)** | **8.9** | **203 (151, 253)** | **7.3** | **241 (187, 295)** | **11.1** |
| Colorectum (C18-C20) | 198 (127, 267) | 4.0 | 107 (68, 144) | 3.8 | 92 (60, 123) | 4.2 |
| Gallbladder (C23) | 8 (6, 11) | 0.2 | 3 (2, 4) | 0.1 | 5 (4, 7) | 0.2 |
| Pancreas (C25) | 83 (17, 148) | 1.7 | 42 (8, 77) | 1.5 | 39 (8, 72) | 1.8 |
| Breast (C50) | 16 (-10, 42) | 0.3 | 0 | 0.0 | 16 (-10, 42) | 0.7 |
| Endometrium (C54.1) | 61 (44, 78) | 1.2 | 0 | 0.0 | 61 (43, 78) | 2.8 |
| Kidney (C64) | 77 (61, 93) | 1.5 | 51 (40, 60) | 1.8 | 27 (21, 32) | 1.2 |
| **Chronic renal failure (N18)** | **135 (63, 204)** | **2.7** | **70 (33, 105)** | **2.5** | **65 (30, 99)** | **3.0** |
| **Liver disease (K70, K74)** | **251 (154, 345)** | **5.0** | **169 (111, 225)** | **6.0** | **83 (44, 116)** | **3.8** |
| *Total deaths prevented under 75* | *1775 (1578, 1978)* | *35.6* | *1235 (1094, 1380)* | *44.1* | *540 (474, 608)* | *24.8* |
| **Total deaths averted or delayed** | **4979 (4446, 5555)** | **100.0** | **2799 (2494, 3124)** | **100.0** | **2180 (1912, 2450)** | **100.0** |
| *Actual number of diet-related NCD deaths in Canada (2019)* | *92845* | | *46568* | | *46277* | |
| ***% of actual diet-related NCD deaths that could be averted or delayed* ^4^** | ***5.4*** | | ***6.0*** | | ***4.7*** | |

| **Table S4.4.** Scenario 4: Food substitution for all *CCHS-Nutrition 2015* adult participants who consumed at least one packaged food product that would display a ‘high in' FOPL symbol | | | | | | |
| --- | --- | --- | --- | --- | --- | --- |
| **Cause of death (ICD-10 Code) ^1^** | **Total (mean, 95% UI) ^2^** | **% ^3^** | **Males (mean, 95% UI) ^2^** | **% ^3^** | **Females (mean, 95% UI) ^2^** | **% ^3^** |
| **Cardiovascular diseases** | **4937 (4199, 5705)** | **70.1** | **2805 (2392, 3217)** | **71.0** | **2140 (1778, 2504)** | **69.1** |
| Ischaemic heart disease (I20-25) | 2470 (2083, 2866) | 35.1 | 1663 (1403, 1919) | 42.1 | 808 (595, 1019) | 26.1 |
| Cerebrovascular disease (I60-69) | 930 (697, 1154) | 13.2 | 435 (331, 537) | 11.0 | 496 (374, 618) | 16.0 |
| Heart failure (I50) | 807 (555, 1056) | 11.5 | 373 (264, 483) | 9.4 | 438 (292, 574) | 14.1 |
| Aortic aneurysm (I71) | 31 (13, 53) | 0.4 | 21 (9, 34) | 0.5 | 10 (4, 17) | 0.3 |
| Pulmonary embolism (I26) | 7 (2, 13) | 0.1 | 3 (1, 7) | 0.1 | 3 (1, 7) | 0.1 |
| Rheumatic heart disease (I05-09) | 5 (2, 11) | 0.1 | 2 (1, 5) | 0.1 | 3 (1, 7) | 0.1 |
| Hypertensive disease (I10-15) | 694 (533, 844) | 9.8 | 308 (239, 377) | 7.8 | 385 (294, 468) | 12.4 |
| **Diabetes (E11, E14)** | **936 (700, 1129)** | **13.3** | **528 (405, 630)** | **13.4** | **410 (299, 500)** | **13.2** |
| **Cancer** | **639 (489, 782)** | **9.1** | **290 (215, 363)** | **7.3** | **348 (271, 428)** | **11.2** |
| Colorectum (C18-C20) | 285 (185, 383) | 4.0 | 153 (99, 205) | 3.9 | 132 (89, 175) | 4.3 |
| Gallbladder (C23) | 12 (8, 16) | 0.2 | 4 (3, 6) | 0.1 | 8 (5, 10) | 0.3 |
| Pancreas (C25) | 118 (24, 209) | 1.7 | 59 (12, 107) | 1.5 | 57 (11, 104) | 1.8 |
| Breast (C50) | 25 (-14, 61) | 0.4 | 0 | 0.0 | 24 (-13, 61) | 0.8 |
| Endometrium (C54.1) | 88 (64, 112) | 1.2 | 0 | 0.0 | 88 (64, 111) | 2.8 |
| Kidney (C64) | 111 (89, 134) | 1.6 | 73 (57, 87) | 1.8 | 39 (31, 47) | 1.3 |
| **Chronic renal failure (N18)** | **189 (87, 285)** | **2.7** | **98 (48, 148)** | **2.5** | **91 (41, 138)** | **2.9** |
| **Liver disease (K70, K74)** | **352 (214, 483)** | **5.0** | **237 (151, 318)** | **6.0** | **115 (60, 162)** | **3.7** |
| *Total deaths prevented under 75* | *2523 (2233, 2822)* | *35.8* | *1754 (1549, 1958)* | *44.4* | *769 (672, 869)* | *24.8* |
| **Total deaths averted or delayed** | **7047 (6249, 7886)** | **100.0** | **3949 (3508, 4403)** | **100.0** | **3099 (2710, 3497)** | **100.0** |
| *Actual number of diet-related NCD deaths in Canada (2019)* | *92845* | | *46568* | | *46277* | |
| ***% of actual diet-related NCD deaths that could be averted or delayed* ^4^** | ***7.6*** | | ***8.5*** | | ***6.7*** | |

1. WHO, International Statistical Classification of Diseases and Related Health Problems, Tenth Revision. 2. 95% UI are based on 10,000 iterations of Monte Carlo analysis built in PRIME. 3. Percentage from total diet-related NCD deaths that could have been prevented or delayed. 4. Percentage of actual deaths in Canada (2019) attributable to the diet-related NCDs under study. Note: total deaths averted or delayed represent less than the sum of the individual diet related NCD mortality causes given that double counting has been accounted for in PRIME during the modelling process. The same applies to the sum of CVDs and cancers.

**Table S5.** Age- and sex-specific estimates of the annual number of diet related NCD deaths in Canada, 2019 (4-8)

| **Males** | **20-24** | **25-29** | **30-34** | **35-39** | **40-44** | **45-49** | **50-54** | **55-59** | **60-64** | **65-69** | **70-74** | **75-79** | **80-84** | **85+** |
| --- | --- | --- | --- | --- | --- | --- | --- | --- | --- | --- | --- | --- | --- | --- |
| I60-I69: Cerebrovascular diseases | 3 | 7 | 14 | 22 | 30 | 72 | 101 | 190 | 280 | 452 | 641 | 829 | 1,018 | 2,288 |
| I20-I25: Ischaemic heart diseases | 2 | 8 | 17 | 65 | 122 | 269 | 573 | 1,049 | 1,631 | 1,999 | 2,324 | 2,394 | 2,691 | 5,626 |
| C00-C14: Lip, oral cavity and pharynx | 2 | 2 | 3 | 4 | 11 | 28 | 53 | 110 | 158 | 158 | 185 | 131 | 86 | 126 |
| C15: Oesophagus | 0 | 0 | 3 | 9 | 17 | 33 | 76 | 158 | 238 | 281 | 273 | 232 | 203 | 198 |
| C16: Stomach | 0 | 4 | 5 | 7 | 12 | 22 | 48 | 91 | 130 | 162 | 175 | 175 | 172 | 241 |
| C34: Bronchus and lung | 2 | 3 | 4 | 12 | 26 | 57 | 211 | 589 | 1,133 | 1,590 | 1,946 | 1,815 | 1,440 | 1,551 |
| C25: Pancreas | 0 | 3 | 4 | 3 | 13 | 39 | 92 | 200 | 334 | 374 | 475 | 429 | 321 | 431 |
| C18-20: Colorectum | 3 | 5 | 22 | 34 | 61 | 104 | 172 | 318 | 440 | 564 | 680 | 613 | 703 | 1,031 |
| C50: Breast | - | - | - | - | - | - | - | - | - | - | - | - | - | - |
| C54.1: Endometrium | - | - | - | - | - | - | - | - | - | - | - | - | - | - |
| C23: Gallbladder | 0 | 0 | 0 | 0 | 1 | 3 | 4 | 6 | 5 | 18 | 15 | 14 | 9 | 19 |
| C64: Kidney | 0 | 0 | 1 | 6 | 8 | 14 | 49 | 89 | 129 | 163 | 191 | 151 | 146 | 225 |
| I10-I15: Hypertensive disease | 0 | 0 | 5 | 6 | 14 | 27 | 58 | 97 | 117 | 136 | 156 | 167 | 244 | 620 |
| E11, E14: Diabetes | 5 | 4 | 13 | 16 | 42 | 64 | 100 | 225 | 294 | 390 | 538 | 526 | 522 | 923 |
| C67: Bladder cancer | 0 | 0 | 1 | 2 | 2 | 11 | 16 | 39 | 90 | 136 | 197 | 215 | 289 | 545 |
| C22: Liver cancer | 1 | 3 | 3 | 10 | 15 | 15 | 67 | 180 | 291 | 367 | 364 | 308 | 246 | 229 |
| C53: Cervix cancer | - | - | - | - | - | - | - | - | - | - | - | - | - | - |
| K70, K74: Liver disease | 0 | 13 | 17 | 35 | 62 | 115 | 188 | 329 | 363 | 417 | 307 | 229 | 172 | 117 |
| I50: Heart failure | 0 | 2 | 1 | 0 | 9 | 4 | 35 | 47 | 95 | 119 | 232 | 293 | 480 | 1,534 |
| I71: Aortic aneurysm | 1 | 1 | 6 | 11 | 12 | 22 | 32 | 41 | 79 | 90 | 141 | 104 | 144 | 260 |
| I26: Pulmonary embolism | 2 | 3 | 2 | 1 | 4 | 10 | 15 | 27 | 22 | 25 | 47 | 43 | 31 | 59 |
| I05-09: Rheumatic heart disease | 0 | 0 | 1 | 2 | 4 | 3 | 5 | 6 | 10 | 9 | 29 | 34 | 35 | 75 |
| N18: Chronic renal failure | 1 | 1 | 0 | 2 | 5 | 8 | 7 | 24 | 42 | 72 | 97 | 152 | 183 | 508 |

| **Females** | **20-24** | **25-29** | **30-34** | **35-39** | **40-44** | **45-49** | **50-54** | **55-59** | **60-64** | **65-69** | **70-74** | **75-79** | **80-84** | **85+** |
| --- | --- | --- | --- | --- | --- | --- | --- | --- | --- | --- | --- | --- | --- | --- |
| I60-I69: Cerebrovascular diseases | 3 | 7 | 16 | 19 | 26 | 56 | 89 | 138 | 221 | 322 | 516 | 746 | 1,044 | 4,494 |
| I20-I25: Ischaemic heart diseases | 1 | 1 | 3 | 14 | 32 | 70 | 142 | 263 | 511 | 717 | 1,067 | 1,216 | 1,845 | 7,230 |
| C00-C14: Lip, oral cavity, and pharynx | 1 | 0 | 1 | 2 | 4 | 12 | 16 | 25 | 47 | 43 | 59 | 63 | 50 | 91 |
| C15: Oesophagus | 0 | 0 | 1 | 1 | 3 | 6 | 13 | 28 | 53 | 64 | 75 | 82 | 56 | 99 |
| C16: Stomach | 0 | 2 | 5 | 5 | 15 | 14 | 29 | 50 | 49 | 80 | 94 | 106 | 112 | 184 |
| C34: Bronchus and lung | 0 | 1 | 4 | 12 | 36 | 74 | 187 | 612 | 1,009 | 1,326 | 1,690 | 1,596 | 1,242 | 1,523 |
| C25: Pancreas | 0 | 1 | 4 | 5 | 12 | 32 | 68 | 154 | 221 | 298 | 374 | 376 | 385 | 565 |
| C18-20: Colorectum | 1 | 1 | 10 | 22 | 56 | 69 | 136 | 192 | 266 | 334 | 430 | 501 | 549 | 1,285 |
| C50: Breast | 1 | 2 | 42 | 62 | 119 | 193 | 350 | 454 | 508 | 570 | 624 | 585 | 579 | 1,179 |
| C54.1: Endometrium | 1 | 0 | 1 | 2 | 9 | 17 | 24 | 66 | 117 | 139 | 164 | 119 | 117 | 128 |
| C23: Gallbladder | 0 | 0 | 0 | 0 | 1 | 0 | 7 | 5 | 10 | 17 | 24 | 27 | 24 | 38 |
| C64: Kidney | 0 | 0 | 1 | 1 | 3 | 8 | 16 | 42 | 41 | 63 | 75 | 79 | 91 | 189 |
| I10-I15: Hypertensive disease | 0 | 0 | 0 | 2 | 7 | 14 | 15 | 27 | 61 | 92 | 124 | 166 | 259 | 1,418 |
| E11, E14: Diabetes | 2 | 3 | 7 | 14 | 14 | 25 | 56 | 112 | 155 | 249 | 308 | 342 | 428 | 1,157 |
| C67: Bladder cancer | 0 | 0 | 0 | 1 | 5 | 7 | 11 | 33 | 21 | 52 | 67 | 95 | 99 | 281 |
| C22: Liver cancer | 1 | 1 | 0 | 5 | 13 | 15 | 35 | 75 | 117 | 142 | 160 | 152 | 180 | 237 |
| C53: Cervix cancer | 2 | 3 | 12 | 25 | 37 | 36 | 42 | 60 | 46 | 44 | 38 | 29 | 20 | 39 |
| K70, K74: Liver disease | 2 | 6 | 17 | 28 | 32 | 65 | 104 | 157 | 172 | 203 | 156 | 141 | 95 | 117 |
| I50: Heart failure | 0 | 2 | 1 | 2 | 3 | 4 | 6 | 26 | 43 | 77 | 159 | 243 | 415 | 2,465 |
| I71: Aortic aneurysm | 0 | 0 | 1 | 1 | 2 | 3 | 7 | 7 | 18 | 38 | 60 | 84 | 109 | 284 |
| I26: Pulmonary embolism | 3 | 2 | 1 | 6 | 8 | 6 | 11 | 18 | 22 | 36 | 45 | 44 | 40 | 115 |
| I05-09: Rheumatic heart disease | 1 | 1 | 1 | 2 | 3 | 3 | 6 | 9 | 12 | 29 | 31 | 51 | 63 | 190 |
| N18: Chronic renal failure | 0 | 4 | 3 | 2 | 1 | 2 | 15 | 14 | 25 | 49 | 79 | 114 | 156 | 552 |

**Table S6.** Age- and sex-specific estimates of the Canadian population, 2019 (9)

| **Age** | **Male** | **Female** |
| --- | --- | --- |
| **20-24** | 1,292,739 | 1,182,807 |
| **25-29** | 1,353,893 | 1,272,165 |
| **30-34** | 1,319,176 | 1,285,925 |
| **35-39** | 1,288,783 | 1,292,390 |
| **40-44** | 1,198,765 | 1,223,278 |
| **45-49** | 1,190,901 | 1,206,950 |
| **50-54** | 1,246,625 | 1,257,812 |
| **55-59** | 1,368,185 | 1,383,192 |
| **60-64** | 1,236,748 | 1,276,958 |
| **65-69** | 1,017,348 | 1,080,436 |
| **70-74** | 817,561 | 890,019 |
| **75-79** | 543,317 | 621,481 |
| **80-84** | 348,047 | 440,404 |
| **85+** | 304,616 | 531,841 |

**References**

1. Health Canada. Table of Reference Amounts for Food (2016) [cited 2021 January 6]. Available from: <https://www.canada.ca/en/health-canada/services/technical-documents-labelling-requirements/table-reference-amounts-food.html>.

2. Statistics Canada. Canadian Community Health Survey – Nutrition: Public Use Microdata File (2019) [cited 2022 October 12]. Available from: <https://www150.statcan.gc.ca/n1/en/catalogue/82M0024X>.

3. Health Canada. 2015 Canadian Community Health Survey - Nutrition. Reference Guide to Understanding and Using the Data (2017) [cited 2022 October 12]. Available from: <https://www.canada.ca/en/health-canada/services/food-nutrition/food-nutrition-surveillance/health-nutrition-surveys/canadian-community-health-survey-cchs/reference-guide-understanding-using-data-2015.html>.

4. Statistics Canada. Table 13-10-0142-01 Deaths, by Cause, Chapter Ii: Neoplasms (C00 to D48) (2019) [cited 2021 January 4]. Available from: <https://www150.statcan.gc.ca/t1/tbl1/en/tv.action?pid=1310014201>.

5. Statistics Canada. Table 13-10-0144-01 Deaths, by Cause, Chapter Iv: Endocrine, Nutritional and Metabolic Diseases (E00 to E90) (2019) [cited 2021 January 4]. Available from: <https://www150.statcan.gc.ca/t1/tbl1/en/tv.action?pid=1310014401>.

6. Statistics Canada. Table 13-10-0147-01 Deaths, by Cause, Chapter Ix: Diseases of the Circulatory System (I00 to I99) (2019) [cited 2021 January 4]. Available from: <https://www150.statcan.gc.ca/t1/tbl1/en/tv.action?pid=1310014701>.

7. Statistics Canada. Table 13-10-0151-01 Deaths, by Cause, Chapter Xiv: Diseases of the Genitourinary System (N00 to N99) (2019) [cited 2021 January 4]. Available from: <https://www150.statcan.gc.ca/t1/tbl1/en/tv.action?pid=1310015101>.

8. Statistics Canada. Table 13-10-0148-01 Deaths, by Cause, Chapter Xi: Diseases of the Digestive System (K00 to K93) (2019) [cited 2021 January 4]. Available from: <https://www150.statcan.gc.ca/t1/tbl1/en/tv.action?pid=1310014801>.

9. Statistics Canada. Table 17-10-0005-01 Population Estimates on July 1st, by Age and Sex (2019) [cited 2021 January 4]. Available from: <https://www150.statcan.gc.ca/t1/tbl1/en/tv.action?pid=1710000501>.
